# Primary care-led weight-management intervention: qualitative insights into patient experiences at two-year follow-up

**DOI:** 10.1080/17482631.2023.2276576

**Published:** 2023-11-20

**Authors:** Marie Spreckley, Judith de Lange, Jaap Seidell, Jutka Halberstadt

**Affiliations:** a Department of Health Sciences, Faculty of Science, Vrije Universiteit Amsterdam, London, UK; b Vrije Universiteit Amsterdam, Amsterdam, The Netherlands

**Keywords:** Obesity, long-term weight loss, obesity management, weight management, weight-loss, sustainable weight-loss

## Abstract

**Purpose:**

The prevalence of overweight and obesity is continuously increasing globally and long-term weight loss intervention outcomes remain disappointing. To determine which behavioural intervention approaches improve the probability of achieving long-term weight loss, this two-year follow-up study aimed to identify distinct factors and strategies for successful long-term weight loss maintenance.

**Methods:**

A cohort of 20 participants with overweight and obesity from a primary-care led weight management programme with diverse backgrounds was interviewed at baseline, after 1 and 2 years, and asked to do quantitative self-description. This study focused on the 2-year follow-up interviews from this study series.

**Results:**

We found that agile, continuous self-monitoring with personalized, sustainable lifestyle habits correlated with positive outcomes. Participants reported health benefits, maintained weight loss, and found motivation in supportive peer networks. Challenges like anxiety, disappointment, and disruptions derailed progress. Long-term success relied on a strong support system of healthcare professionals, friends, and family.

**Conclusions:**

The findings of this study series highlight the intricate nature of long-term weight loss maintenance. This study corroborates the persistence of overarching themes while highlighting the individual variability in their relative importance. Findings emphasize the importance of long-term support to effectively address the diverse needs of patients trying to achieve long-term weight loss maintenance.

## Introduction

The global prevalence of overweight and obesity is continuously rising with estimates predicting that 1 in 5 adults globally will have obesity by 2025 (World Obesity, 2021). Alongside this trend, 13% of all global healthcare expenditure is expected to be spent on obesity related co-morbidities (World Obesity, 2021). Overweight and obesity can result in an array of health impairments both physically and mentally. The risk of developing weight related co-morbidities is usually correlated with the degree of excess weight, yet genetic and epigenetic predispositions also play a significant part (Pi-Sunyer, 2009). Having overweight or obesity has been shown to increase the likelihood of developing 13 types of cancer along with increasing the likelihood of developing type 2 diabetes (T2D), cardiovascular disease, hypercholesterolemia, non-alcoholic fatty liver disease, osteoarthritis, sleep apnoea and gastrointestinal complications, amongst others (Cancer Research UK, 2020; Pi-Sunyer, 2009). Individuals with overweight or obesity also have an increased likelihood of developing anxiety and depression, which is often a result of experienced discrimination based on their weight and appearance (Emmer et al., 2020). Weight stigma is omnipresent including places of work, healthcare settings and social settings, making it hard to escape and disengage (Emmer et al., 2020).

Even though the mechanisms resulting in overweight and obesity may appear to be a simple imbalance in energy intake compared to energy expenditure, the potential underlying drivers are many, varied and individualized. Contributing factors include social, economic, psychological, physiological, biological and infrastructural determinants, amongst others. Particularly the complex interaction between individuals and environmental factors often explains different experiences in weight trajectories (McGlashan et al., 2018). Due to the complex causes associated with overweight and obesity, the quest to find the optimal, sustainable weight loss approach has been exhaustive. Studies have repeatedly demonstrated that a variety of dietary approaches work equally well both for weight loss and adherence if a consistent calorie deficit is achieved (Adegboye & Linne, 2013; Franz et al., 2007; Hall & Guo, 2017; Ostendorf et al., 2019; Tzur et al., 2018; Wadden et al., 2015). Unfortunately, while short-term weight loss can appear promising, the majority of dieters regain their lost weight within a year, often eventually resulting in a higher weight than at baseline (Dulloo et al., 2012; Maclean et al., 2018). Notably, individuals who manage to maintain their weight loss for more than 2 years have significantly better likelihoods of maintaining their weight loss over the coming 5 to 10 years (Natvik et al., 2018).

Due to the disappointing long-term outcomes demonstrated by behavioural interventions for significant, long-term weight loss maintenance, it became apparent that the experiences patients encounter when trying to achieve long-term weight loss require further investigation. In the United Kingdom (UK), primary care offers a unique setting for weight management interventions due to its accessible nature, resulting in a multitude of studies investigating the most promising, scalable approaches in this setting (Aveyard et al., 2016; Jolly et al., 2011; Madigan et al., 2022). Qualitative research is increasingly being utilized to examine the diverse experiences of individuals with overweight or obesity in their pursuit of long-term weight loss maintenance. However, there is a notable lack of longitudinal studies focusing on this aspect, as evident from reviews of qualitative studies on weight management interventions (Garip et al., 2016; Greaves et al., 2017; Hartmann-Boyce et al., 2014; Spreckley et al., 2021). To help bridge this gap, we conducted a longitudinal, primary care-based interview series to gain insights into the essential factors and strategies associated with successful long-term weight loss maintenance within a primary care-led weight-loss intervention. This article presents the findings of a two-year follow-up (24 months) study conducted as part of an annual interview series conducted at baseline, 12 months and 24 months by the authors, which specifically focused on the participants’ perspectives. The aim of this study was to explore the subjective experiences of individuals participating in a primary care-led weight-management intervention over two years. By examining the complexities and nuances of sustainable weight loss efforts, this study aimed to identify factors and strategies for successful long-term weight loss maintenance. The implications of these insights intend to inform individuals with obesity, professionals involved in obesity management services and behavioural weight-management researchers.

## Materials and methods

Qualitative methods were chosen to gain an understanding of participants’ experiences and the contextual factors that shape their weight loss journeys (Lincoln & Guba, 1985). Grounded in the interpretivist paradigm, this study recognizes that individuals construct their own realities through subjective interpretations (Green & Thorogood, 2018). The interpretivist paradigm emphasizes the subjective experiences and understandings of individuals, positing that reality is socially constructed and best understood through deep, qualitative exploration of these subjective perspectives (Health Data Research UK, 2023).

### Context of the study

The study was conducted within the setting of a National Health Service (NHS) primary care weight management intervention in London, United Kingdom. The intervention focused on supporting patients in achieving long-term weight loss through education and assistance in various aspects, including nutrition, physical activity, stress management, sleep hygiene and overcoming everyday challenges. The intervention involved a multidisciplinary team of clinicians, including nutritionists, dietitians, medical doctors and psychotherapists, who provided support via video, phone and an app. The programme structure allowed for flexibility to accommodate individual circumstances and enhance long-term adherence and success. The multidisciplinary team collaborated with the patients’ General Practitioners throughout the programme. Successful, long-term weight loss maintenance was defined as having achieved and maintained ≥ 10% intentional weight loss between baseline and 2-years since successful long-term weight loss is frequently defined as consistently maintaining intentional weight loss of more than 10% for over a year (Montesi et al., 2016). Further details about the programme can be found in the one-year follow-up study by the authors (Spreckley et al., 2023).

### Research Team

The research team consisted of specialists in obesity (MS, JS, JH), weight management (MS, JS, JH), psychology (JH), health sciences (JdL, JS), public health (JS, JH) and clinical practice (MS). The lead author (MS) was a senior nutritionist in the primary care practice this study series was conducted in and was responsible for developing a behavioural weight management protocol for patients with overweight, obesity and weight-related co-morbidities. The team’s diverse expertise ensured the safety of the intervention and access to medical assessments. The lead author had established working relationships with all the participants.

### Recruitment and participants

Participants were purposefully recruited for the baseline study and all, but one, agreed to participate in the interview for this two-year follow-up study. Due to various COVID-19 restrictions, all interviews were conducted remotely via audio, as chosen by the participants. Recruitment criteria included being an adult, having a BMI >25 kg/m^2^, fluency in English, absence of diagnosed eating disorders and clearance from a medical doctor at the practice. The participants represented a diverse group in terms of backgrounds, experiences and perspectives related to weight loss maintenance in a clinical setting. By intentionally recruiting a diverse sample, the study aimed to capture a wide range of perspectives to comprehensively explore the complex factors influencing weight loss maintenance. This two-year follow-up cohort (Table I and Table II) consisted of 12 female and 8 male participants (61.9% female, 38.1% male) between the ages of 34 and 72 (mean age 47.62), some of which had weight related co-morbidities (Table II). BMIs ranged from 22.4 kg/m^2^ to 47.4 kg/m^2^ (average BMI 38.84 kg/kg/m^2^) and self-reported ethnicities included 10 White British/European participants, 4 Asian British participants and 6 African/Caribbean British participants (52.38% White, 19.05% Asian and 28.57% Black) (Table I). Patient 13 did not participate in the two-year interview due to personal reasons.

**Table I.** Participant characteristics at baseline, 1-year and 2-year follow-up.

#	Gender	Ethnicity	Age	BMI Start	BMI Y1	BMI Y2	Kg Start	Kg Y1	Kg Y2	KG 0–2	Programme
1	female	Caucasian	45	42.6	42.9	43.9	119	120	122.5	−2.9%	Option 2
2	female	Caucasian	34	36.6	32.9	36.3	106	95	105	0.9%	Option 2
3	male	Black	46	30.6	25.6	26	96	81	82	14.6%	Option 1
4	male	Asian	42	33.8	28.8	31	100	85.1	92	8.0%	Option 1
5	female	Caucasian	45	28.7	24.9	28.8	83	72	83.4	−0.5%	Option 2
6	male	Black	36	32.1	30.2	32.7	116.6	109.7	117.9	−1.1%	Option 2
7	male	Black	35	33.5	30	28.7	106.1	95	91	14.2%	Option 2
8	female	Black	62	40.5	35.6	37.5	107	94	98.5	7.9%	Option 1
9	female	Caucasian	67	31.2	27.1	27.4	82	70	72	12.2%	Option 2
10	male	Caucasian	72	27.7	23.4	24.1	84.2	69	72.5	13.9%	Option 2
11	female	Black	56	29	24.2	24.9	84	70	73	13.1%	Option 2
12	female	Asian	43	29.4	25.5	27.2	74.5	64.5	69	7.4%	Option 1
13	female	Caucasian	34	43.3	43		111	110			Option 2
14	female	Asian	47	36.1	29.9	29.6	77	63.7	63.1	18.1%	Option 1
15	male	Black	56	48.4	47.3	47.4	136	133	133.5	1.8%	Option 1
16	female	Caucasian	42	36.6	25.4	22.4	101	70	62	38.6%	Option 2
17	male	Caucasian	46	39.8	32.1	32.2	120	104.4	105	12.5%	Option 2
18	female	Caucasian	69	29	26.2	26.9	69	63	64.5	6.5%	Option 1
19	male	Caucasian	68	44.2	34.5	34.8	138	109	109.5	20.7%	Option 2
20	female	Caucasian	39	44	44.5	46.4	103	104.5	108.7	−5.5%	Option 2
21	female	Asian	37	37.6	34.9	34.3	94.1	87	85.7	8.9%	Option 2
**Range**	**13 F, 8 M**	**11 C, 4 A, 6 B**	**34–72**	**27.7–48.4**	**23.4–47.3**	**22.4–47.4**	**69–138**	**63–133**	**62–133.5**	**-0.1% − 0.4%**	**7 O1, 14 O2**

**Table II.** Participant co-morbidities and medications at baseline, 1-year and 2-year follow-up.

#	Co-morbidities Start	Co-morbidities Y1	Co-morbidities Y2	Medications/Support Start	Medications/Support Y1	Medications/Support Y2
1	IBS, gallstones, chronic pancreatitis, GORD	IBS, gallstones, chronic pancreatitis, GORD	IBS, gallstones, chronic pancreatitis, GORD	Omeprazole (GORD)	Omeprazole (GORD)	Omeprazole (GORD)
2	Infertility, chronic knee pain	Infertility, chronic knee pain	Infertility, chronic knee pain, anxiety	None	None	Sertraline (anxiety)
3	T2D, hypertension,hypercholesterolemia	T2D, hypertension,hypercholesterolemia	T2D, hypertension, hypercholesterolemia	Atorvastatin (hypercholesterolemia), amlodipine(hypertension)	Atorvastatin (hypercholesterolemia), amlodipine (hypertension)	Atorvastatin (hypercholesterolemia), amlodipine (hypertension)
4	T2D, hypertension	T2D	T2D	Metformin (T2D), losartan (hypertension)	None	None
5	Depression	Depression	Depression	Prozac (depression)	Prozac (depression)	Prozac (depression)
6	Severe sleep apnoea	Medium sleep apnoea	Medium sleep apnoea	CPAP machine (sleep apnoea)	CPAP machine (sleep apnoea)	CPAP machine (sleep apnoea)
7	Severe GI complications	Mild GI complications	Very mild GI complications	None	None	None
8	T2D, hypertension,hypercholesterolemia, arthritis	Hypertension, arthritis	Hypertension, arthritis	Metformin (T2D), atorvastatin (hypercholesterolemia), furosemide (hypertension)	Furosemide (hypertension)	Furosemide (hypertension)
9	Colon cancer, hypertension, asthma	Colon cancer, asthma	Asthma	Beloc zok cor (hypertension), symbicort inhaler (asthma)	Symbicort inhaler (asthma)	Symbicort inhaler (asthma)
10	Hypercholesterolemia, asthma, arthritis	Hypercholesterolemia, asthma, arthritis	Hypercholesterolemia, asthma, arthritis	Simvastatin (hypercholesterolemia), symbicort inhaler (asthma)	Simvastatin (hypercholesterolemia), symbicort inhaler (asthma)	Simvastatin (hypercholesterolemia), symbicort inhaler (asthma)
11	Hypertension	Hypertension, gastritis	Hypertension, gastritis	None	None	None
12	T2D, gastritis, hypertension, IBS	Prediabetes, hypertension	T2D, hypertension	Metformin (T2D), losartan (hypertension)	Metformin (prediabetes), losartan (hypertension)	Metformin (prediabetes), losartan (hypertension)
13	Depression, anxiety, arthritis	Depression, anxiety, arthritis		None	None	
14	Prediabetes, GORD	GORD	None	None	None	None
15	T2D, hypertension, COPD	T2D, hypertension, COPD	T2D, hypertension, COPD	Metformin (T2D), atorvastatin (hypercholesterolemia), doxazosin (hypertension), losartan (hypertension), aspirin (blood clots), amlodipine (hypertension)	Metformin (T2D), atorvastatin (hypercholesterolemia), doxazosin (hypertension), losartan (hypertension), aspirin (blood clots), amlodipine (hypertension)	Metformin (T2D), atorvastatin (hypercholesterolemia), doxazosin (hypertension), losartan (hypertension), aspirin (blood clots), amlodipine (hypertension)
16	None	None	None	None	None	None
17	asthma	asthma	asthma	Symbicort inhaler (asthma)	Symbicort inhaler (asthma)	Symbicort inhaler (asthma)
18	T2D, hypertension, arthritis, GORD	Hypertension, arthritis	Prediabetes, hypertension, arthritis	Metformin (T2D), Omeprazole (GORD), felodipine (hypertension), naproxen (arthritis)	Felodipine (hypertension), naproxen (arthritis)	Felodipine (hypertension), naproxen (arthritis)
19	GORD, arthritis, hypertension	Arthritis	Arthritis	Lansoprazole (GORD), Ibuprofen (arthritis), amlodipine (hypertension)	Ibuprofen (arthritis)	Ibuprofen (arthritis)
20	PCOS, hypertension, depression	PCOS, hypertension, depression	PCOS, hypertension, depression, kidney, bladder and GI problems (not diagnosis yet)	Metformin (PCOS)	Metformin (PCOS)	Metformin, Valsartan, Gabapentin Tillomed, Amlopidine, Metifene
21	PCOS, hypertension, IBS	PCOS, hypertension, IBS	Hypertension, IBS	Metformin (PCOS), ramipril (hypertension)	Metformin (PCOS), ramipril (hypertension)	Ramipril (hypertension)
	**Patients with co-morbidities: 20**	**Patients with resolved co-morbidities: 10**	**Patients with resolved co-morbidities: 11**	**Patients with medications/support: 15**	**Patients using less medications/support: 6**	**Patients using less medications/support: 6**

## Method of data gathering

Semi-structured interviews were conducted by the lead author to obtain rich and detailed data from participants regarding their experiences, perspectives and strategies related to weight loss maintenance. Participants were interviewed at baseline, at 12 months and at 24 months. This study focused on the findings from the 24-month interviews, yet took into account the findings of the previous interviews at baseline and 12 months. Individual interviews were chosen to delve into participants’ personal experiences, motivations and challenges (Green & Thorogood, 2018). The interview topics covered participants’ experiences in achieving weight loss, personal strategies and facilitators, motivators and obstacles, self-perception and the role of their environment. The duration of the interviews ranged from 22 minutes to 37 minutes. All interviews were audio-recorded, transcribed verbatim and accompanied by reflective writing in a logbook by the lead author.

In addition to conducting interviews, we incorporated a visual Likert scale tool (Jebb, 2021) as part of our data collection process. Participants were presented with this tool prior to the interviews and verbally responded to it during the sessions. The visual Likert scale facilitated participants’ self-description of their current weight, goal weight, and overall experience, providing a standardized and structured approach to gathering their self-reported information. This enabled us to quantitatively analyse and compare their responses. At the start of the treatment, participants’ weight was measured at the medical centre, and they subsequently recorded their own weight using the provided app. Additionally, the metabolic markers of patients with type 2 diabetes were monitored every three months. We also included quantitative data to document the following individual participant data over time: weight, metabolic markers, conditions and medications. This facilitated further, individual longitudinal analysis (Table I–IV).

**Table III.** Participant Weight Loss % Year 1 and Total.

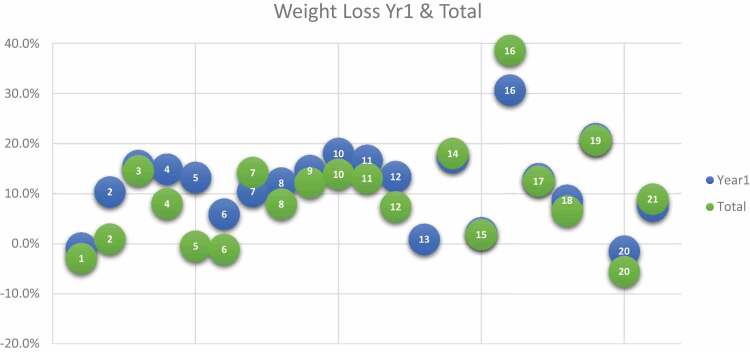

Successful, long-term weight loss maintenance was defined as having achieved > 10% intentional weight loss at Year 1 and Year 2/Total (Total in Green).

**Table IV.** Weight Loss Targets and Achievements.

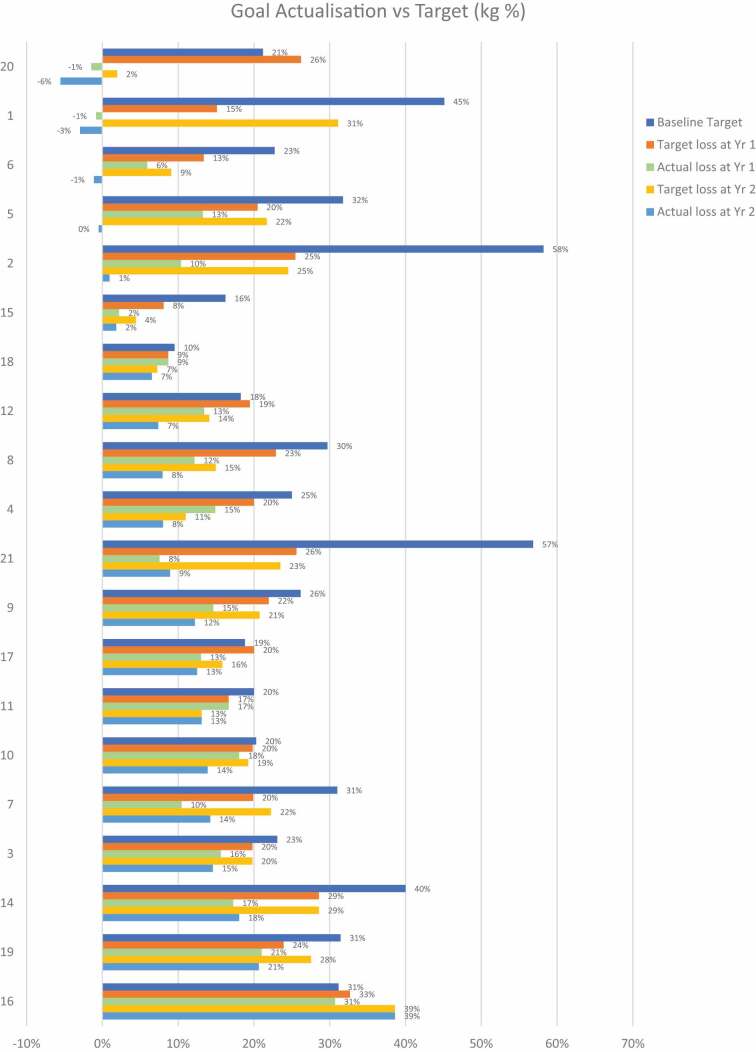

Initial 1 year weight loss target % at baseline, actual weight loss % Year 1, 2-year weight loss target % at Year 1, actual weight loss % Year 2, 3-year target weight loss % at Year 2.

### Data analysis

In order to capture the intricate nature of individual circumstances and account for the multidimensional aspects of this longitudinal study series, we utilized template analysis (Brooks et al., 2014) to incorporate the themes discovered in the systematic review, baseline study and one-year follow-up by the authors (Spreckley et al., 2021, 2022, 2023). As new themes emerged, they were added to the code tree. After each interview, the lead author listened to the recordings again and wrote short summaries, which were reviewed by the second, third and fourth author. Once all interviews were completed, the transcripts were externally transcribed and the lead and second authors independently conducted open line-by-line coding utilizing template analysis to allow for the creation of a comparable data-set (King, 2004). New codes were added as they emerged. The researchers compared and discussed all categories with the fourth and fifth authors until a consensus was reached to minimize biases arising from individual backgrounds. The agreed-upon themes were identified and suitable quotes were selected for each theme. Since this is a 2-year follow-up study, this approach enabled a comprehensive longitudinal analysis, including the observation of changes, trends and outcomes over time. This comprehensive approach created a deeper understanding of complex interactions that may not be apparent in short-term or cross-sectional studies. Data analysis was facilitated using a combination of computerized (Atlas.ti) and manual techniques.

### Ethical considerations

Participants were provided with comprehensive verbal and written information about the interview format, content, suitable setting, and time requirements. They were informed that certain topics might be challenging and were encouraged to conduct the interviews in a private and comfortable environment. Interview schedules were tailored to accommodate participants’ work and family commitments, with interviews predominantly scheduled in the afternoons and evenings. Participation was voluntary and anonymous, and participants were provided with a detailed consent form, contact information of the research team and information about the affiliated institution. Additionally, they were given contact details to report any negative experiences or outcomes. All participants provided both oral and written consent. The study was approved by the ethics review committee of the Faculty of Science (BETHCIE), Vrije Universiteit Amsterdam, the Netherlands.

### Patient and public involvement and engagement (PPIE)

Our PPIE approach (Health Data Research UK, 2023) was structured the following way:

Study Design Input: Prior to finalizing the study design, a preliminary consultation was conducted with a subset of potential participants and stakeholders. This was to ensure the research questions, methods and expected outcomes resonated with the needs and priorities of those with overweight and obesity. Their feedback directly influenced the final study design.

Questionnaire Development: When designing the quantitative self-description component of the study, we actively sought feedback from a PPIE group consisting of individuals who had firsthand experience with weight management challenges. This approach ensured that our questions were relevant, sensitive and comprehensible.

Interview Process: Our interview methodology was also shaped by PPIE. We piloted our interview questions with several volunteers from our target demographic. Their feedback allowed us to refine the questions for clarity, remove potentially triggering content and include topics that might not have been considered initially by the research team.

Interpretation of Results: After gathering the data, a subset of participants was involved in focus group discussions to help interpret the findings. This ensured that our interpretations aligned with the lived experiences and perspectives of those we studied.

Feedback Loop: Once preliminary results were obtained, they were shared with a PPIE group and HCPs for validation. Any discrepancies between the researchers’ interpretations and the group’s perspectives were discussed and resolved.

Dissemination Strategy: To ensure our findings reached both the academic community and the public effectively, our dissemination strategy was crafted in collaboration with patients and HCPs. This helped in making our findings accessible and understandable to a broader audience, and in selecting the best platforms and forums for sharing the results.

Support System Recommendations: Our conclusions, particularly regarding the importance of a support system, were influenced by the consistent feedback from our PPIE interactions. The emphasis on the diversity of needs stemmed directly from the personal stories and experiences shared with us throughout the research process.

## Results

### Weight loss targets and achievements

The majority of participants did not achieve their baseline and 1-year weight loss targets (Table III and IV). Participant 16 was an exception and managed to achieve her 31% body weight loss target at year 1, then adjusted her target to 39% at year 1 and achieved this at year 2. Most participants decreased their weight loss target at year 1, yet did not achieve this at year 2. Successful weight loss was defined as having achieved an intentional reduction in weight of more than 10%. Table IV presents the initial weight loss target percentage from baseline, the actual weight loss percentage at Year 1, and the weight loss target from baseline for Year 2. Notably, the majority of participants found experiencing various degrees of weight loss and resulting benefits motivating.

“Obviously, the weight loss was the most rewarding and building confidence, feeling better about myself. Feeling that I wanted to go out”. [female, White, 45y - P5]

“I think my confidence has improved significantly due to the weight loss and holistically”. [female, Black, 56y - P11]

“I felt lighter, I started to walk more, challenge myself, it gives you more confidence”. [male, Back, 56y - P15]

**Figure 1.**
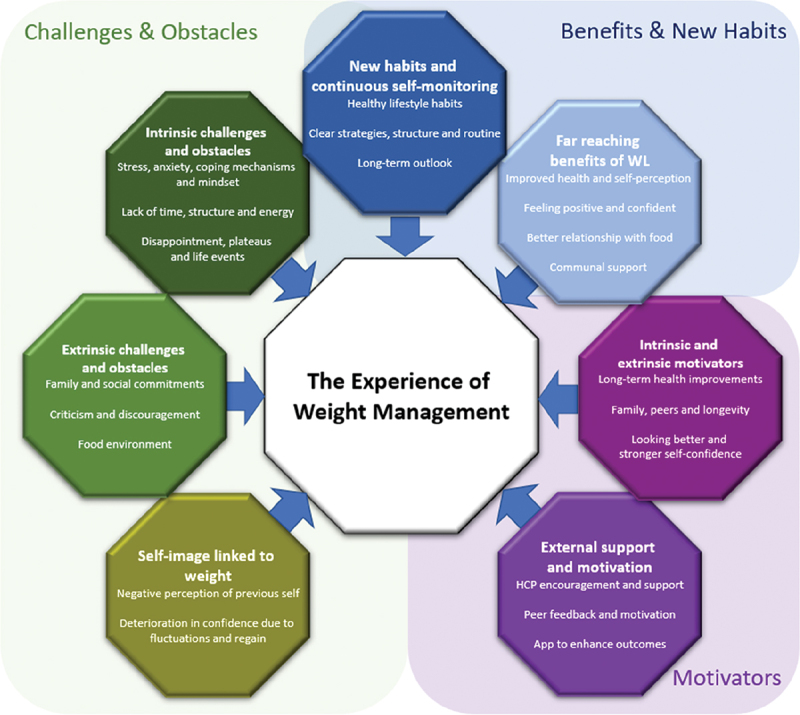
The experiences of weight management.

Upon analysis of the 2-year data, it became apparent that the main themes capturing participant experiences after one year continued to persist in the second year (Figure 1). However, it also became evident that the significance and impact of these themes varied individually (Appendix). This observation emphasized that while the overarching challenges remained unchanged, their specific manifestations differed for each individual, highlighting the unique nature of their experiences (Appendix).

The themes confirmed in this study included: 1) motivators, which consisted of intrinsic and extrinsic motivators, external support and motivation; 2) challenges and obstacles, which consisted of individual challenges and obstacles, environmental challenges and obstacles and self-image linked to weight; and 3) benefits and new habits, which consisted of the wide-ranging benefits of weight loss, the development of new habits and continuous self-monitoring (Figure 1).

The following sections will present an in-depth explanation of each theme, supported by representative quotes. Each quote will be accompanied by the corresponding respondent number, which can be cross-referenced with the participant numbers listed in Table I and II.

## Motivators

### Intrinsic and extrinsic motivators

Motivators frequently described were both intrinsic and extrinsic in nature. Participants expressed wishing to attain and maintain better health to live longer, more fulfilled lives. They also described wishing to be healthy long-term for their families and close peers. The desire to feel better looking and achieving stronger self-confidence was also frequently discussed. These motivators became more solidified across the whole cohort in the second year for participants who maintained and regained the lost weight. Having experienced some weight loss even with eventual regain provided glimpses of hope and motivation. Intrinsic and extrinsic motivators can be summarized as: 1) long-term health improvements, 2) family, peers and longevity, and 3) looking better and stronger self-confidence.

Firstly, many participants expressed their desire to enhance their overall health and decrease their reliance on medications. A common motivator for weight loss was the aspiration to alleviate weight-related conditions and to improve general mobility for the future. Many were driven by the goal of reducing specific health issues directly associated with their weight. Improving overall health and well-being served as a powerful incentive for most participants to embark and continue on their weight management journeys. By focusing on these objectives, they sought to enhance their quality of life and maintain optimal, long-term health.

“If I get hit by a bus, that’s one thing, but if I die of a heart attack, that’s something else I could have potentially have changed. It’s little things like that, that you start thinking about too”. [male, Black, 46y - P3]

“I think the fact that I don’t take any pills anymore actually. Because obviously I stopped taking all my pills and everything seems to be all right, I’m going downwards. I think that was a big motivation both years to not having to go back to taking pills again”. [male, Asian, 42y - P4]

“Even though I still got the arthritic knees and hips and that, but I can do more than what I could before. That is motivation in itself”. [male, White, 68y - P19]

“I think if I hadn’t have done this, well, I know, if I hadn’t have done this, my mobility would have been compromised hugely”. [female, White, 69y - P18]

Secondly, family members still played a significant role and served as strong motivators for most participants on their weight management journeys. Many participants found inspiration in wanting to be a positive influence on their family members, consciously serving as a source of motivation for their loved ones. Additionally, some participants shared that their own weight loss achievements acted as a source of inspiration for their peers, encouraging them to embark on their own health journeys. Furthermore, some participants expressed a strong desire to increase their chances of living longer, driven by the aspiration to be present for important life events and experiences. This sense of motivation stemming from familial connections and personal relationships served as a driving force in their commitment to achieving long-term weight loss goals.

“I can’t continue like this. It’s just the realization of Christmas. I need to be here for my son, I need to be here for me, I need to be here for my husband. If I keep going the way I am, I’m not going to live to a very long age, am I? It puts it into perspective”. [female, White, 45y - P1]

“To increase the motivation was that I knew that if I maintain a good weight, then I would motivate my wife to do the same and my children and grandchildren to understand that not being overweight was important”. [male, White, 72y – P10]

“I motivated lot of people as well to lose weight. Whenever I see some staff, people who have known me in the past, they can’t believe it and they will stop me. They stop and ask me for my tips. I love feedback”. [female, Asian, 43y - P12]

“My new goal is to be able to run after my child and stick around long enough to teach him a few things”. [male, Black, 36y - P6]

Lastly, some participants had specific life events they wished to appear their best for, such as weddings or special occasions, while others found joy in the simple pleasure of being able to fit into a wider range of clothing options. Many more successful participants experienced notable improvements in self-confidence as a result of their weight loss journey and this newfound confidence played a critical role in maintaining their motivation. Moreover, this increased confidence also translated into feeling better equipped to effectively manage their weight in the future. The enhanced self-assurance and positive self-perception stemming from weight loss achievements empowered participants in their ongoing pursuit of a healthier lifestyle.

“This is always about looking good for my wedding, essentially”. [male, Black, 36y - P6]

“My motivation, if anything, improved in the second year, because I’d already gained momentum and carried on seeing the great results, in terms of how I felt, how I looked, the clothes that I could fit into”. [female, Black, 56y - P11]

“I think I’m more confident now. I’m more self-appreciating myself. I’m considering more my well-being and my body, which I didn’t have time before that so much”. [female, White, 67y - P9]

“What motivated me to stay on track, these feelings. I’m good about myself. I’m confident. It seemed great”. [female, White, 45y - P5]

### External support and motivation

Participants consistently emphasized the significance of receiving external support and encouragement from HCPs and peers as a critical element in achieving long-term success. External support and motivation can be summarized as: 1) Healthcare Practitioner (HCP) encouragement and support and 2) peer feedback and motivation. Interestingly, participants did not feel that the app was a necessary tool to help enhance their efforts in the second year as their own routines and approaches were more solidified.

Firstly, both WLMs and regainers expressed the value of ongoing support from HCPs, while peer feedback and motivation played a pivotal role in increasing adherence and drive. Words of encouragement from HCPs were particularly impactful in enhancing adherence for weight loss maintainers, which proved to be of great importance, especially during the challenges imposed by the pandemic.

“It was essential because I did not want to disappoint my nutritionist. The feedback I got, which is very encouraging, solidified my wish to maintain the lower weight”. [male, White, 72y – P10]

“Actually, having someone being supportive and saying like, ‘You have done it, be proud of yourself and confident in your own skin,’ it’s really good to hear that. It makes you focus on the right things rather than unachievable goals”. [female, White, 34y - P2]

“You gave me a lot of support throughout those pandemic months. […] Your advice, your friendship over the last two years, has been invaluable to me and my family, so I mean it”. [male, Black, 46y - P3]

Secondly, participants often experienced feedback and encouragement from their peers and HCPs, which significantly enhanced their motivation. The support they received from family members, including their children, was also a recurring theme, with their loved ones providing frequent words of encouragement. The combined positive feedback from peers, HCPs and family members served as powerful sources of motivation, reinforcing their commitment to their weight loss journey and instilling a sense of pride in their accomplishments.

*“Oh, people have been really happy for me to be quite honest with you. They’ve been quite encouraging, compliments and even when we do things and so on*. [female, Black, 62y—P8]

“Oh, they’re absolutely thrilled to bits. My daughter, she keeps saying, ‘I can’t believe how small you look, mom’.” [female, White, 69y - P18]

“Well, my friends have been amazed. They say it’s amazing that I’ve kept it off and everything like that. Family has been really good and been amazed. The kids have been amazed. For example, I had to go to see my GP and he hadn’t seen me for over two years, he didn’t even know it was me”. [female, White, 42y - P16]

The app was utilized significantly less by all participants in the second year and some participants also found new apps to help them monitor their progress.

*“The app’s really good as well.”* [female, White, 45y—P1]

“I wanted to eat, I had an app, which was actually for a keto diet, but it actually worked out calories in meal plans”. [female, White, 45y - P5]

## Benefits and new habits

### Solidified new habits and continuous self-monitoring

During the second year of the program, participants commonly established and solidified new habits and self-monitoring strategies, including continuously tracking, modifying and remaining mindful of food consumption patterns. New habits and continuous self-monitoring can be summarized as: 1) healthy lifestyle habits, 2) clear strategies, structure and routine, 3) long-term outlook.

Firstly, both participants who maintained and regained the lost weight described successfully adapting to and reaping the benefits of a variety of healthy lifestyle habits, complemented by effective self-monitoring strategies. These included adopting healthier and more mindful eating practices, incorporating regular physical activity and implementing strategies to reduce stress levels. Particularly successful WLMs emphasized the importance of allowing themselves time to develop a balanced and sustainable approach, finding the process itself to be encouraging and motivating. These newly embraced healthy habits served as anchors during challenging times and aided participants in managing emotional eating. “Honestly, at the beginning, it was a little bit of a challenge modifying your behaviour. As I got into it, things improved, and it was actually a gradual process which was really good. I think I really benefited from that program because it just taught me how to create a balance in what I eat and how to modify my diet. In fact, I’ve maintained the weight, which is great”. [female, Black, 56y - P11]

“Obviously, the loss of weight itself, which is the result of my newly adoptive behaviour itself is very rewarding and motivating”. [female, Asian, 43y - P12]

“I find the toughest part is the motivation, but because I got into a routine, it became like second nature”. [male, Back, 56y - P15]

“Now, I deal with things in a different way and I don’t use food as a comfort for that”. [male, White, 46y - P17]

“It was a game-changer, which became a habit, a positive habit”. [male, White, 72y – P10]

Secondly, continuously prioritizing intentionality in the utilization of strategies for consistency and accountability emerged as recurring themes, particularly among more successful participants. Frequently utilized approaches included thorough preparation and planning, allowing individuals to effectively navigate their weight management journey. Establishing regular exercise schedules played a critical role in boosting motivation and yielding positive outcomes. Additionally, staying well-informed and mindful about the ingredients and calorie content of foods consumed contributed to achieving sustained success. By implementing these strategies, participants were able to enhance their level of control and make informed choices that aligned with their goals.

“I think just sticking to my regime and staying focused mentally and everything, I think that will help me maintain it”. [female, White, 42 y - P16]

“There was that lack of discipline, but now there a structure there from the morning, and that is, I said it, quite healthy”. [male, Back, 56y - P15]

“I think I try to produce meals, which are very different, variable, and so it’s not getting boring, and this I think it contributes to my being able to keep my weight down and that of my partner. […] Amazingly, I’m very aware of calories and I’m checking everything that I’m buying, but I have the time. I must say I have the time to look at the whole picture and I count the calories”. [female, White, 67y - P9]

“I exercise three, four, five times a week now.” [female, Black, 62y—P8]

Thirdly, many participants expressed having adopted a well-balanced, long-term perspective on their weight management journey, which was a particularly emphasized by more successful participants. They described having a clear understanding of the necessary steps needed to achieve their goals and to maintain long-term success. This newfound perspective empowered them, providing a sense of control and confidence in their ability to navigate the challenges that arise along the way. By embracing this long-term mindset, participants felt equipped to make sustainable lifestyle changes and sustain their progress over time.

“I’ve realized that to sustain that, I have to sustain the lifestyle changes all around. Not just eating habits, exercise, looking at what I’m actually eating, and having a mixed and varied diet as opposed to just one diet to suit all”. [female, White, 45y - P5]

“I think my ideal plan, to be honest, will be to continue the exercise and just to do it that way bit by bit by bit. It’s not going to be something that happens overnight. I think there’s that realization as well, weight loss isn’t— and healthy eating, it isn’t a trend. It isn’t something that you do for a short period of time. It’s got to be a lifestyle change”. [male, Black, 46y - P3]

“I understand now what that’s all about. It’s not only what you eat, it’s how you approach what you eat and around it all. It’s not just going without, losing a bit and then go back to what you were. No, it’s not. It is a life of change and you have to keep going with it”. [female, White, 69y - P18]

### Long-term wide-ranging benefits of weight loss

Most more successful participants described a multitude of benefits associated with weight loss including improved health from baseline and the 1-year follow-up, improved self-perception and enhanced feelings of positivity and confidence. Many also described having a better relationship with food, and peers were frequently perceived as supportive and encouraging. Notably, regainers felt the strongest fluctuations in these categories once they regained lost weight. The wide-ranging benefits of weight loss can be summarized as: 1) improved health and self-perception, 2) feeling positive and confident, 3) better relationship with food, and 4) social support.

Firstly, more successful participants described experiencing rapid and significant health improvements as a result of weight loss, including heightened energy levels and a reduction in weight-related co-morbidities. Some also believed that reducing excess weight decreased their vulnerability to contracting COVID-19. Participants described that successful weight loss had a positive impact on their mental health as well, leading to a sense of improved overall well-being. They described the benefits of an enhanced self-perception, including increased confidence in their ability to effectively manage their weight.

“I felt great, healthier, more energetic. I wasn’t coming home at night, falling asleep”. [female, White, 42y - P16]

“That feeling coming back up of being a bit thinner and feeling a lot healthier, and not tired and no achy knees and stuff like that was a really nice thing”. [female, White, 34y - P2]

“All those things affect you mentally, psychologically as well so I feel more confident in myself”. [female, Black, 56y - P11]

“I think just feeling good in my own skin, not embarrassed to take my T-shirt off on the beach when we went on holiday. Just feeling good about what I look like. That helps you keep motivated on the good behaviours because you feel better in your own skin”. [male, White, 46y - P17]

Secondly, a significant number of participants expressed a sense of increased happiness and positivity compared to their previous self. For some, the impact of their weight loss journey was truly life-changing, resulting in a sense of reinvention and the ability to experience new opportunities, including enjoying the shopping, wearing bathing suits and getting a chance to live life to its full potential. Confidence improvements were consistently reported among weight loss maintainers (WLMs) and participants commonly mentioned that their weight loss made daily tasks easier to manage. Overall, participants described a general feeling of enhanced well-being, attributing it largely to the achievement of weight loss maintenance.

“I just feel happier in myself which is great.” [female, Black, 56y—P11]

“Well, you have that extra spring in your step. You think faster. You don’t feel lethargic. I haven’t felt lethargic for a long time to be honest”. [male, Back, 56y - P15]

*“And feel like I’m living life I don’t know, more to the full of my life really. […]I think a lot more of myself and I’m more confident in myself and in going out and I don’t mind—I feel a lot more confident. I have to go swimming. I work with a special needs school; we go swimming with the children. I feel more comfortable now within myself when I have to get into a costume or anything like that.”* [female, White, 42 y—P16]

“I feel confident walking out the door every day, and I love going shopping. I love getting new clothes”. [female, Asian, 47y - P14]

“I feel better about myself having lost the weight. Much better. It’s probably better than last year because I’ve lost it and kept it off”. [male, White, 68y - P19]

Thirdly, participants shared the benefits experienced from adapting new portion sizes and embracing different dietary patterns. They described a sense of adaptability and flexibility in their approach to portion control, finding it manageable to modify their eating habits. Participants were pleased to discover that making changes to their portion sizes and overall dietary patterns was not as daunting or difficult as previously anticipated. This newfound understanding empowered them to continue their weight management journey with optimism and a greater sense of control over their eating habits.

“Very quickly, we adapted to it and our bodies adapted to it. What we found, I guess quite surprising is now I can’t eat a big meal anymore”. [male, Black, 36y - P6]

“I have absolute—I don’t miss any specific types of food. I actually just really enjoy the diet that we do have. I think that’s quite surprising. I mean, we never ate massively badly, but I mean, there are less frequent bad days as it were or cheap things as it were. It’s not because of the fact that I’m denying myself. It’s just because of the fact that actually I’m enjoying eating lots of other things, which I find quite nice”. [male, Black, 35y - P7]

“We’ll just eat our normal meals, careful watching what we eat. Saturday, we’d probably have something extra, but we control everything. We find it easy to do that. Saturday or Sunday, we’ll have a pudding, have something nice over the weekend, and then the rest of the week, we’re careful of what we have, and then, what we’ll eat”. [male, White, 68y - P19]

Lastly, peers were consistently perceived as a source of support and encouragement by participants. They described the joy of receiving uplifting words and compliments, which were deeply motivating. External recognition and validation played a significant role in reinforcing their progress and boosting their confidence along their journey. The positive interactions with peers further solidified the importance of social support in their pursuit of weight loss maintenance.

*“Oh, they’re absolutely thrilled to bits.”* [female, White, 69y—P18]

“I love feedback and it’s good that I’ve got a supportive staff and a good environment because it helps you a lot for being positive thinker. It helps you maintain your weight. Then be a good example to them. I love it. I love this attention”. [female, Asian, 47y - P14]

“Oh, people have been really happy for me to be quite honest with you. They’ve been quite encouraging, compliments and even when we do things and so on.” [female, Black, 62y—P8]

## Challenges and obstacles

### Intrinsic challenges and obstacles

Challenges participants encountered were very varied and dependent on individual circumstances. Participants experienced diverse levels of stress and faced often challenging individual life events. This frequently triggered a loss of structure, anxiety, frustration and disappointment. COVID-19 was one of the most notable aspects discussed by regainers in relation to this category. Notably, participants had gained a lot more clarity around how challenges and obstacles impacted their success after having been mindful of their behaviours for two years. Intrinsic challenges and obstacles can be surmised as: 1) stress, anxiety, coping mechanisms and mindset, 2) lack of time, structure and energy, and 3) disappointment, plateaus and life events.

Firstly, participants frequently expressed experiencing stress when faced with changing work environments due to COVID-19, as well as job changes and redundancies, leading to a sense of uncertainty. In response to this uncertainty, many individuals found themselves resorting to familiar coping mechanisms, such as seeking comfort from food or turning to alcohol. Furthermore, mindset changes also played a role in decreasing adherence to their weight management goals.

“Then my new job is so full on that I’ve put my job in front of my wellbeing. I can definitely tell you that I was doing nine-hour a day taking no breaks, going to notebooks, so I had no sun time either after two years that we worked from home. I was very much sat at a desk all the time”. [female, White, 45y - P5]

*“When you’re uncertain, you drink and eat.”* [male, Black, 36y—P6]

“You stay in your pyjamas and your track suits. You eat basic rather than you can actually cook something particularly nice it becomes—because you just get into a bit of a rough and a low”. [female, Black, 62y - P8]

“All things with the stress, you naturally come home and uncork a bottle of wine. Before you know it, that’s gone and then the next day just feeling rubbish. […] Live the day, really. Yes. No, there was no focus at all”.” [female, White, 34y - P2]

Secondly, participants often found the lack of time resulting from changing commitments a notable challenge. This lack of time not only affected their ability to adhere to their previously established exercise routines but also disrupted their overall sense of structure. The absence of a structured routine often led participants off course and decreased their overall adherence to their weight management goals. These challenges posed significant obstacles in their efforts to maintain consistency and stay on track with their desired routines.

“I think again working 15, 16-hour days at times, it’s just all those learned behaviours just compared to when it became convenient, eating late at night and stuff like that”. [female, White, 34y - P2]

“I don’t have time to go to the gym.” [female, Asian, 47y—P14]

“The last 12 months have been horrendous. We haven’t been following any diet, any sort of plan. We’ve just been completely off script, completely falling off the wagon compared to the first 12 months”. [female, White, 45y - P1]

Thirdly, participants expressed a decrease in motivation and an increase in frustration, often attributed to experiencing a plateau or stagnation in their weight loss progress. These periods of slowed or halted weight loss presented challenges to WLMs and affected their overall sense of motivation. Furthermore, participants identified social situations, particularly those involving alcohol, as triggers that made weight loss maintenance more challenging. Navigating social events where food and drink choices were abundant and potentially less healthy posed difficulties for participants in adhering to their weight management goals.

“It feels good that I’ve maintained but it feels bad because I couldn’t lose more, I think it’s because I expected a bit more from myself”. [female, Asian, 37y - P21]

“I’m a bit disappointed is that I wanted to lose some more but then, we haven’t out to come over to the gym for a time”. [male, White, 68y - P19]

“Barbeque and beer are what I turn to in my moments of need or to help my mental health. Barbeques and some beer”. [male, Black, 36y - P6]

“It’s never really about food. It’s always about the drink”. [female, Black, 62y - P8]

Experiencing weight regain frequently evoked feelings of disappointmentand a sense of failure among individuals. Alongside the challenges of weight regain, participants also encountered difficult life events such as moving to a new location, health problems and the loss of a loved one. These events brought additional emotional and practical hurdles to their weight management journey.

”As I said, I’m ashamed I’ve let it go this far. I never thought I would. I always promised myself, “Now I’ve lost all the weight, I’m not going to put it back on, I’m going to be really good.” I’m a bit ashamed that it didn’t take much to tip me over the edge and just become—I thought I had more willpower than that, so I’m a bit embarrassed about myself.” [female, White, 45y—P1]

“Because of the relocation, new house, set everything up and then just after that we were going home so we completely stopped cooking at home. That was another thing that changed my behaviour that after moving we stopped cooking”. [female, Asian, 37y - P21]

“I’ve obviously had this kidney, whatever’s going on with that area going on. I’ve had the bladder issue, I’ve had the bowel issue, so I’ve had a lot going on”. [female, White, 39y - P20]

## Extrinsic challenges and obstacles

Extrinsic challenges including family and social commitments, along with criticism and discouragement, often hindered weight loss efforts. The food environment and unexpected circumstances also impacted success Extrinsic challenges and obstacles can be surmised as: 1) family and social commitments, 2) criticism and discouragement, and 3) food environment.

Firstly, balancing home-schooling with other responsibilities proved to be a challenge for many participants as the demands of education and personal commitments intertwined. Additionally, some individuals had the added responsibility of supporting family members who were dealing with health problems, which further stretched their resources and time. Social occasions posed challenges to adhering to their weight management goals, as these events often involved temptations and unhealthy food choices. The holiday season, including Christmas and other holidays, presented a particular test of willpower, as participants navigated gatherings and traditions cantered around festive foods and drinks. Successfully maintaining adherence during these occasions required significant effort and determination.

“How am I going to cope with this now? He’s never going to be here. Home school is all going to be me”. [female, White, 45y - P1]

“My daughter’s got ADHD and she’s on the autistic spectrum and she was really struggling. She’d been struggling at school for a long time. […] It just went from bad to worse”. [female, White, 39y - P20]

“I need to avoid those external influences to sometimes stay on track because it is sometimes a bit easy to fall off the bandwagon because of, ‘Oh, it’s Friday,’ or, ‘Oh, it’s a nice day outside’.” [male, Black, 36y - P6]

“Then Christmas came, and it was just like massive overindulgent.” [female, White, 45y - P5]

Secondly, participants often faced criticism from their peers, which was equally common among both WLMs and regainers. They experienced discouraging remarks about their appearance and had their chosen approach questioned by others. These criticisms from peers had a negative impact on their self-esteem and added an additional layer of difficulty to their weight management journey. The unsupportive comments and questioning of their methods underscored the importance of creating a supportive and understanding environment to foster successful weight management.

“Family, friends, it’s been the same, ‘You’ve put on a bit weight,’ so not really encouragement, more negative comments”. [female, White, 45y - P1]

*“I’ve had people say “Oh, you’re wasting away, don’t lose any more.”* [female, White, 42 y—P16]

*“You hit some roadblocks where some people say to you, “Oh, you’ve lost a lot of weight, are you unwell or?”* [female, Black, 56y—P11]

“Some people make you feel: ‘You’ve lost too much weight, are you eating healthily,’ so you do encounter that as well, which was to a degree quite challenging and off-putting, but you just need to overcome that”. [male, Asian, 42y - P4]

Lastly, the availability of take-away food substantially increased during and after lockdowns, resulting in heightened consumption, often driven by feelings of boredom. This accessibility contributed to participants indulging in more frequent food intake. In an effort to combat temptation and enhance adherence to their weight management goals, some participants strategically attempted to avoid environments with readily available food options. By consciously minimizing exposure to such food environments, they aimed to reduce the likelihood of succumbing to temptations and maintain better adherence to their weight management strategies.

“It’s easier to order takeaways every day because you’re not just going out. I think before we went home, we were ordering takeaways three, four times a week, which was way more than our usual”. [female, Asian, 37y - P21]

*“I think it was just that we were locked up so much and it just—I think it was definitely boredom, just sitting down watching telly, and then, “Oh, let’s get a takeaway.”* [female, White, 45y—P5]

“I knew that if I stayed in my safe zone of the house, I didn’t have the temptation or the freedom to go crazy. Whereas if I was out more then I need a— I’m not the sort of person that goes out for one drink or two drinks, as you well know”. [male, Black, 36y - P6]

“Going out was one of the challenges I found, especially when you socialize with people but when it’s at home, I find it’s a lot easier because you don’t have any of those temptations even though it’s all right”. [female, White, 42 y - P16]

## Self-image linked to weight

Participants found it challenging to reflect on their past lifestyle choices and experienced this to be a deterrent to weight loss maintenance. Self-image linked to weight can be surmised as: 1) negative perception of previous self, and 2) deterioration in confidence due to fluctuations and regain.

Firstly, the profound impact of weight changes on self-image and the swift deterioration in confidence and self-perception were frequently expressed. Participants acknowledged that they had not been mindful of their behaviours prior to entering the weight management program. Some participants also believed that their weight gain was a result of a lack of discipline and were critical of their previous lifestyle choices. The process of self-reflection highlighted the need for a shift in mindset and a deeper understanding of the factors that contributed to weight regain.

“In the past I wasn’t thinking about it. I’d be like, ‘Why have I put on weight?’ or whatever because I wasn’t aware. I wasn’t cognizant of my actions and the subsequent consequences that they would have.[…] That’s where historically over the last 5, 6, 7, 8, 10 years that’s how the problem has slowly built up with my weight and stuff because that was a lot of constantly always being out and then always drinking and then always making bad food choices and decisions based off of drinking”. [male, Black, 36y - P6]

“There was that lack of discipline, but now there is structure there from the morning, and that is, I said it, quite healthy”. [male, Black, 56y - P15]

Secondly, weight fluctuations and regain frequently led to a decrease in self-confidence among participants, accompanied by feelings of disappointment and shame. Many expressed being upset with themselves as a result. These negative emotions often resulted in participants temporarily disengaging from the programme, leading to a loss of contact and connection. The impact of weight fluctuations on self-confidence and subsequent disengagement from the programme highlighted the emotional toll and challenges faced by individuals on their weight loss journeys.

“I’m ashamed I’ve let it go this far. I never thought I would. I always promised myself, ‘Now I’ve lost all the weight, I’m not going to put it back on, I’m going to be really good.’ I’m a bit ashamed that it didn’t take much to tip me over the edge and just become - I thought I had more willpower than that, so I’m a bit embarrassed about myself”. [female, White, 45y - P1]

“Cross in the way that I wasn’t able to maintain it, but I also had to give myself a break in that sense and say, ‘I can’t do everything. I have to let something go.’ Unfortunately, I think, in hindsight, I shouldn’t have let that go because as I said before, eating badly drinking, and stuff like that, it just makes everything else harder. I was actually not doing myself a favour at all”. [female, White, 34y - P2]

“I was very focused the first 12 months. It was through your support. I think I distanced myself from the programme when I felt like I was failing”. [female, White, 45y - P5]

## COVID-19

This two-year study commenced three months prior to the first national COVID-19 lockdown in the UK, which included lockdowns at various stages as well as a far reaching direct and indirect consequences triggered by the pandemic. This affected participants in varying ways. Less successful participants generally felt that COVID-19 was significantly more hindering and derailing, while more successful participants felt that it did not have an impact or was more manageable or beneficial in both years. Notably, all participants felt that COVID-19 had a less significant impact on their experience during the second year. The impact of COVID-19 can be surmised as: 1) hindering or derailing, 2) manageable or beneficial.

Firstly, many participants faced challenges in maintaining motivation due to ongoing restrictions and found it particularly difficult to cope with feelings of loneliness and frustration. The combination of home schooling and remote work posed an additional hurdle, as individuals struggled to effectively manage their responsibilities. As a result, some turned to food for entertainment, leading to a pattern of regular indulgence. The prevailing uncertainty experienced throughout both years was also a source of anxiety and concern for participants.

“I just felt that, what’s the point? We can’t go anywhere; we can’t do anything”. [female, White, 39y - P20]

“I very much spent a huge amount of time on my own. It was very unhealthy, I think, just for any human wellbeing. I’d phone my mom every day, but it’s not the same”. [female, White, 45y - P5]

” I was just like, ”‘Oh my God, what am I going to do?’” [female, White, 45y - P1]

“Eating replaced a lot of other sorts of entertainment and everything because of the lockdown and everything. That’s a behaviour that once you’ve been set into that, the only thing to celebrate is to eat. It’s difficult to come out of that”. [female, Asian, 37y - P21]

“The first year and the second year were just full of uncertainty of not really knowing what the situation was going to be like”. [male, Black, 36y - P6]

Conversely, WLMs expressed finding the impact of COVID-19 manageable or not impactful as participants frequently highlighted that, despite the challenges posed by the pandemic itself, it did not negatively impact their ability to maintain their weight loss. Notably, some participants found that focusing on their new lifestyle habits during the pandemic served as a welcome distraction from the uncertainties and disruptions caused by COVID-19. Many participants appreciated the reduced exposure to social temptations, which facilitated better adherence to their weight management goals.

“It’s not affected it. I’ve stayed the same weight now for about a year”. [male, White, 46y - P17]

“Yes, I think it was definitely a challenge to me, the diet wasn’t. That was okay. I focused on that and I think because we made such fun out of it, the COVID part, for a time, I could forget about it in some ways”. [female, White, 69y - P18]

“Compared to last year, I think I don’t see any much difference. We have now got used to it”. [female, White, 67y - P9]

“In a really weird way, it’s probably been good for me because I’ve gone out less. Let me say a couple years ago, I told you that I would drink most nights. Now, we only drink on a Friday”. [female, Black, 62y - P8]

## Individual variations in experiences

The themes that emerged amongst the participants of this cohort were heterogeneous and diverse, which highlights the necessity of individualized support. It became apparent that, even though participants were offered the same programme and support and encountered similar COVID-19 restrictions, the individual variance in experience remained. This was strongly related to personal circumstances and the resulting impact on their ability to engage with the programme. Notably, the programme offered to this particular cohort was highly personalized and was frequently identified as a factor for success by the majority of more successful participants.

“I’m not sure if I wouldn’t have had her as a guidance in the second year. I think I might have lost control mainly when I was sick or when there were happenings, or as Christmas to celebrate, or any other feasts which I was looking for”. [female, White, 67y - P9]

“We transitioned, I think, from nutritionist to therapist.” [male, Black, 36y—P6]

## Discussion

### Main findings

This study set out to explore the experience of trying to achieve weight loss maintenance after having completed the second year of a weight management programme with a particular focus on more successful participants. Determining success enhancing and derailing aspects based on personal accounts of patients has the potential to inform and aid in the optimization of treatment protocols to enhance long-term outcomes. This study is building on a series of studies by the same authors including a systematic review, a baseline study and a 1-year follow up study with the same participant cohort (Spreckley et al., 2021, 2022, 2023). The discovered themes remained in line with the 1-year follow up themes, yet the importance and emphasis changed for all individuals throughout the cohort to varying degrees during the second year of the programme. The themes remained very heterogeneous throughout the cohort, highlighting the unique experiences individuals go through when trying to achieve weight loss and weight loss maintenance. This 2-year follow up study provides valuable insights for HCPs, researchers and patients to gain a deeper understanding of the patient experience and optimize the required behavioural interventions for enhanced, long-term outcomes.

### Comparison with other findings in the literature

A significant amount of research into optimal, short-term weight loss strategies has been conducted, yet sustained, long-term outcomes remain disappointing (Marchesini et al., 2016). Participants frequently manage to engage well with a multitude of short-term intervention approaches, however, when confronted with longer-term, daily complexities including work, family and social situations, the likelihood of remaining on track decreases significantly (Marchesini, et al., 2016). Due to the individual nature of personal, continuously altering experiences, as also determined in this research series, behavioural interventions need to remain agile to facilitate a long-term mindset for optimal, sustained outcomes (Greaves et al., 2017; Hall & Kahan, 2021; Spreckley et al., 2021; Marchesini, et al., 2016).

In the UK, the accessible nature of primary care and unique ability to provide routine monitoring via a multidisciplinary team underscore its potential significance in effectively administering weight management programmes. Primary care settings often have a holistic understanding of patient histories, backgrounds, cultures and lifestyles, allowing primary care providers to offer tailored interventions that resonate with individual needs. The continuity of care, enhanced by trust, facilitates ongoing, open dialogue on sensitive issues including weight and weight related co-morbidities, while multidisciplinary teams commonly found in these settings ensure a comprehensive approach to weight management (Aveyard et al., 2016; Jolly et al., 2011; Madigan et al., 2022). This was also evident in the findings of our longitudinal study series.

The unique contribution of this study series lies largely in its longitudinal nature. Longitudinal analysis can aid in effectively capturing the dynamic nature of experiences, uncovering underlying patterns and trends that contribute to a more comprehensive understanding of participant experiences (Rehackova et al., 2019). This approach revealed nuanced insights and provided a deeper understanding of the intricacies that unfold over time. The findings of this study series show that, while the overarching themes that encapsulate the experience of trying to achieve long-term weight-loss maintenance are largely explored, they change in importance and emphasis for individuals over time due to continuously changing, individual circumstances (Appendix). Longitudinal research into this agile, nuanced experience accompanied by the provision of long-term, continuous support offers the ability to delve deeper into these individual experiences. This can provide a clearer picture of the possible potency weight management interventions of this kind can have on a scalable, population-wide level.

Few qualitative, longitudinal studies have explored the complexities of long-term weight management. Rehackova et al. (2019) conducted a one-year longitudinal qualitative evaluation to investigate behaviour change during a type 2 diabetes remission intervention (Rehackova et al., 2019). The study emphasized the important role of personal beliefs, social influences and environmental factors, highlighting the significance of social support and self-regulation. Personal beliefs, including self-efficacy and control perception, were found to be critical for maintaining healthy behaviours, while social influences and environmental factors, such as support networks and access to healthy options, contributed to successful behaviour change maintenance (Rehackova et al., 2019). Similarly, Thom et al. (2020) conducted a two-year longitudinal qualitative study on weight loss maintenance (Thom et al., 2020). The findings highlighted the challenges individuals face in achieving sustainable weight loss long-term, including cycles of commitment and relapse. Difficulties in maintaining motivation, adherence to healthy behaviours and various barriers such as social pressure, emotional triggers and environmental influences were discovered (Thom et al., 2020). These studies, in line with the findings of the present study, underscore the importance of ongoing support and tailored interventions to address individual needs and experiences in long-term weight loss maintenance.

Considering these findings, along with the insights gained from the systematic reviews by Greaves et al. (2017) and the present authors (2021), it becomes evident that developing strategies that enhance sustained behaviour change are imperative for effective long-term outcomes (Greaves et al., 2017; Spreckley et al., 2021). Self-regulation, environmental factors and comprehensive, continuous support emerge as key elements requiring attention in interventions. These findings align with the recommendations of Kwasnicka et al. (2016), who identified self-regulation, social support and environmental factors as significant in sustaining behaviour change over time (Kwasnicka et al., 2016). Their proposed interventions focus on developing self-regulatory skills, creating supportive environments and integrating social support networks (Kwasnicka et al., 2016. In line with these recommendations, Rothman proposed the Theory of Behavioural Maintenance (TBM), which integrates elements from various behaviour change theories, suggesting that behaviour maintenance is influenced by personal, social and environmental factors (Rothman, 2000). Rothman also emphasizes the importance of self-regulation in maintaining behaviours, including motivation, self-efficacy and goal-setting, as well as the significance of social support in reinforcing behaviour maintenance (Rothman, 2000). Similarly, Sniehotta et al. (2014) highlighted the significance of psychological factors, self-regulation and social support in weight loss maintenance interventions (Sniehotta et al., 2014). Their research emphasizes the need for interventions targeting motivational processes, self-regulation skills, behaviour-specific self-efficacy and addressing emotional factors, coping strategies and positive body image for effective weight loss maintenance (Sniehotta et al., 2014). Addressing the above outlined factors, along with providing comprehensive, continuous support, has the potential to significantly enhance beneficial, long-term outcomes.

### Strengths and limitations

One of the main strengths of this study is that this demographically diverse patient cohort, consisting of varying BMIs, genders, age groups and ethnicities, was followed by the same research team over two years with almost no loss of follow-up. Supporting and interviewing the same patients for this duration provided valuable insights into the unique, longitudinal experiences of trying to achieve weight loss maintenance beyond one year and enabled the creation of a comparable, comprehensive, qualitative dataset. The intervention utilized also provided insights into the second year following an evidence-based weight management programme in primary care, which is a duration less frequently explored. The interviewer directly supported all participants from on-boarding to completion at two years and had a well-established, trusted, individual relationship with each participant, which enhanced open communication and strengthened research engagement.

This can, however, also be interpreted as a potential limitation, as this relationship might have resulted in some participants providing socially desirable responses (Webber et al., 2010). Therefore, the dual role of the researcher also being the main HCP needs to be taken into account when interpreting the experiences and themes discovered (Braun & Clarke, 2019). To enhance self-awareness and critical examination of biases as well as minimize potential bias, the main author engaged in reflexivity by practicing reflective writing to scrutinize assumptions, values and preconceptions that may have influenced data interpretation, leading to a more nuanced and rigorous analysis (Frambach et al., 2013; Silverman, 2016). Notably, we did a longitudinal comparison of the entire cohort yet did not conduct an individual comparison of each participant due to resource constraints, which can be seen as a limitation. We did, however, compare more successful and less successful participants to provide a comprehensive picture. A further limitation may be that the programme and all interviews had to be conducted electronically since this study series was conducted during COVID-19 restrictions, which may have influenced perceptions and experiences when compared to programmes examined via different modalities. Since patients described their own perceptions and experiences, memory recall bias may also have influenced participant perceptions (Ross et al., 2017). Due to the outlined strengths and limitations, studies of this kind need to be considered taking into account the outlined aspects.

### Implications for research and practice

Further research into the most beneficial, scalable treatment strategies taking into account the psychological, physiological and environmental context of each patient will likely aid in enhancing tailored intervention approaches. Scalability remains challenging due to the significant financial investment required, yet the continuously increasing prevalence of overweight and obesity as well as related direct and indirect costs, which have been estimated to be £6.1 billion annually in NHS healthcare costs and £27 billion annually in the impact of economic development in England, make further research into this area vital (Public Health England, 2020b). This might also aid in determining the cost-effectiveness of high-quality, evidence-based care. Some participants expressed being open to participating in group support, which has been shown to facilitate improved, long-term weight loss outcomes for many (Public Health England, 2020a). Further research into scalable yet agile and individualized group support options can inform cost-effective, potent treatment protocols. Notably, a significant number of patients expressed finding individual, one-on-one support instrumental for success both in terms of weight loss and quality of life improvements. Therefore, research into enhancing patient self-efficacy both through behavioural support as well as practical application may prove beneficial, particularly for long-term, sustained weight loss outcomes (Avery, 2018; Jackson et al., 2013; Puhl et al., 2016). Additionally, research into strengthening self-esteem, irrespective of weight loss, may also enhance adherence and motivation and decrease the occurrence of emotional eating episodes, weight fluctuations and regain (Nezami et al., 2016; Smolak, 2001).

The importance of having continuous, personalized HCP support has consistently been found to be beneficial for patient outcomes in the literature (Jackson et al., 2013; Nezami et al., 2016; Scott et al., 2004). This study and others of its kind highlight the importance of taking a comprehensive, longer-term approach with patients due to diverse, continuously changing circumstances and requirements, heavily influenced by both internal experiences and challenging external environments. HCPs working in weight management could benefit from receiving continuous training in behavioural interventions that can enhance long-term weight loss outcomes, especially as research in this field is continuously evolving. Training in self-efficacy enhancing approaches with an emphasis on increasing HCP empathy and agility has the potential to improve long-term treatment outcomes for patients (Nezami et al., 2016; Smolak, 2001). It is also advisable to provide further training and education to HCPs not directly involved in weight management treatment, including GPs, nurses and healthcare advisors, since they often spend a significant amount of time treating patients with weight-related co-morbidities. Helping practitioners enhance their understanding of the patient experience and providing them with practical strategies and tools to support patients effectively may improve long-term weight-loss and quality of life outcomes. The organization and financing of this type of care will likely help optimize treatment effectiveness, including the resolution of weight related co-morbidities.

## Conclusion

This study highlights the intricate dynamics associated with the experience of trying to achieve significant long-term weight loss maintenance over a two-year period. It confirms the enduring presence of overarching themes while shedding light on the individual variations in their relative significance. Importantly, the findings emphasize the inadequacy of short-term interventions and support, as ongoing challenges continually emerge within the unique context of the multifaceted human experience. More successful participants were able to draw on individualized, agile self-monitoring strategies and developed sustainable lifestyle habits, which enhanced success. Supportive peer networks including family, peers and HCPs, served as strong motivators and enhanced long-term adherence. Stress, anxiety, lack of energy and time, often triggered by unanticipated social and environmental factors, frequently challenged adherence and derailed less successful participants. Devising strategies to navigate challenging, continuous intrinsic and extrinsic challenges improved long-term outcomes. This study series highlights that treatment protocols need to take into account the individual experiences, circumstances and requirements of participants and remain agile and flexible as changes occur. Providing continuous, personalized support incorporating well-established, success-enhancing treatment strategies has the potential to enhance long-term outcomes for patients with overweight or obesity.

## Supplementary Material

Supplemental Material

## Appendix

Theme prevalence remaining participants

These tables illustrate how frequently the theoretical themes were discussed by participants with differing genders, BMIs, ethnicities, ages and outcomes broken down into percentages out of a total of 100% to determine the prevalence of each theme during individual interviews. Due to variability in responses, recollections and communication style, the prevalence shown in these graphs serves solely illustrative purposes.

Please note: Patient 13 did not participate in the interviews for this study and patient 2 did not participate in the interviews for the previous 1-year follow-up study due to personal reasons. Blue = Year 1, Green = Year 2

Intrinsic and extrinsic motivators = A, External support and motivation = B, New habits and continuous self-monitoring = C, Far-reaching benefits of WL = D, Intrinsic challenges and obstacles = E, Extrinsic challenges and obstacles = F, Self-image linked to weight = G, COVID-19 = H
