# Supplementary material for: Primary care-led weight-management intervention: qualitative insights into patient experiences at two-year follow-up
Source: Int J Qual Stud Health Well-being. 2023 Nov 20;18(1):2276576. doi: 10.1080/17482631.2023.2276576 (PMC11007633; doi:10.1080/17482631.2023.2276576)

## Appendix

### Theme prevalence remaining participants

These tables illustrate how frequently the theoretical themes were discussed by participants with differing genders, BMIs, ethnicities, ages and outcomes broken down into percentages out of a total of 100% to determine the prevalence of each theme during individual interviews. Due to variability in responses, recollections and communication style, the prevalence shown in these graphs serves solely illustrative purposes.

Please note: Patient 13 did not participate in the interviews for this study and patient 2 did not participate in the interviews for the previous 1-year follow-up study due to personal reasons. Blue = Year 1, Green = Year 2

Intrinsic and extrinsic motivators = A, External support and motivation = B, New habits and continuous self-monitoring = C, Far-reaching benefits of WL = D, Intrinsic challenges and obstacles = E, Extrinsic challenges and obstacles = F, Self-image linked to weight = G, COVID-19 = H


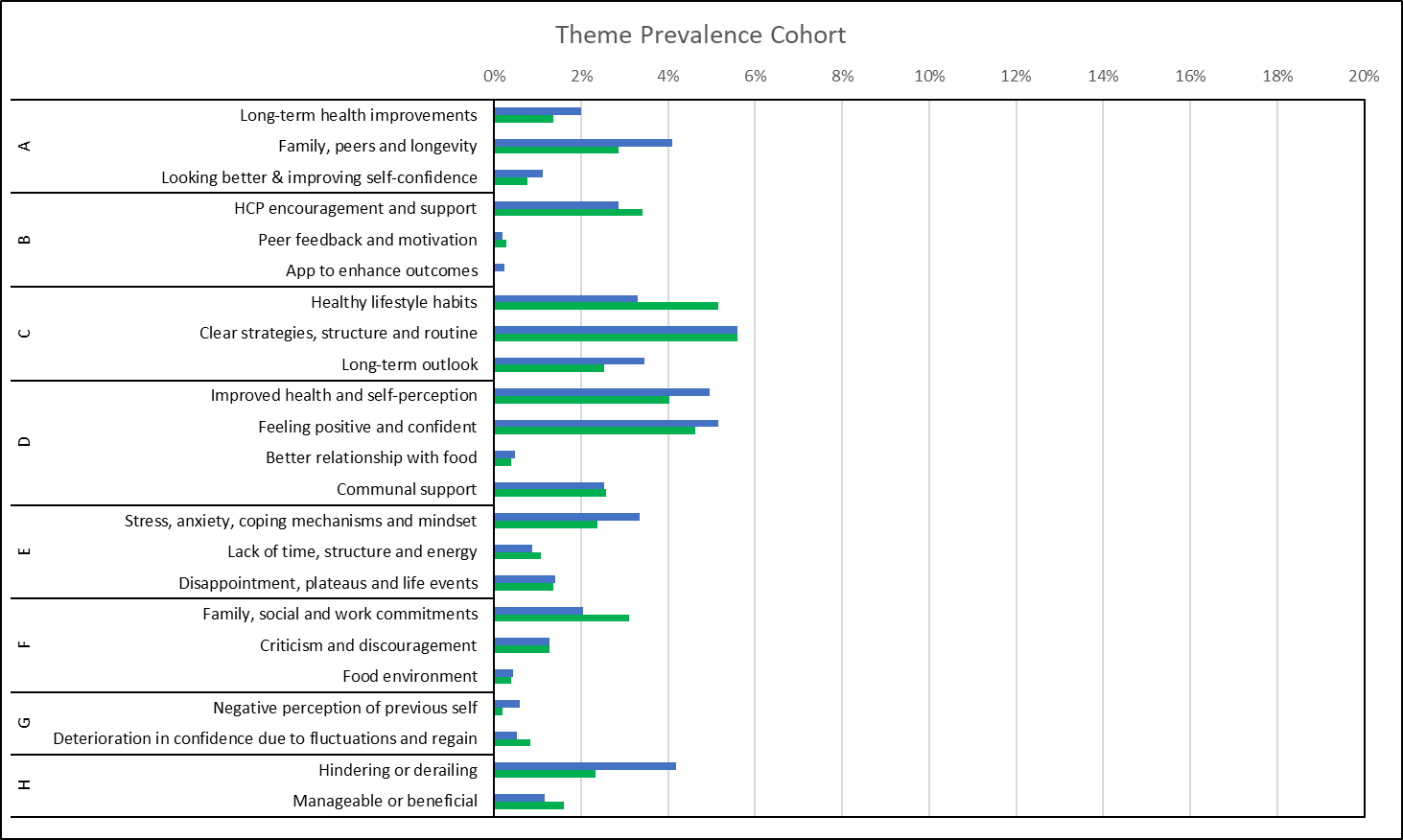


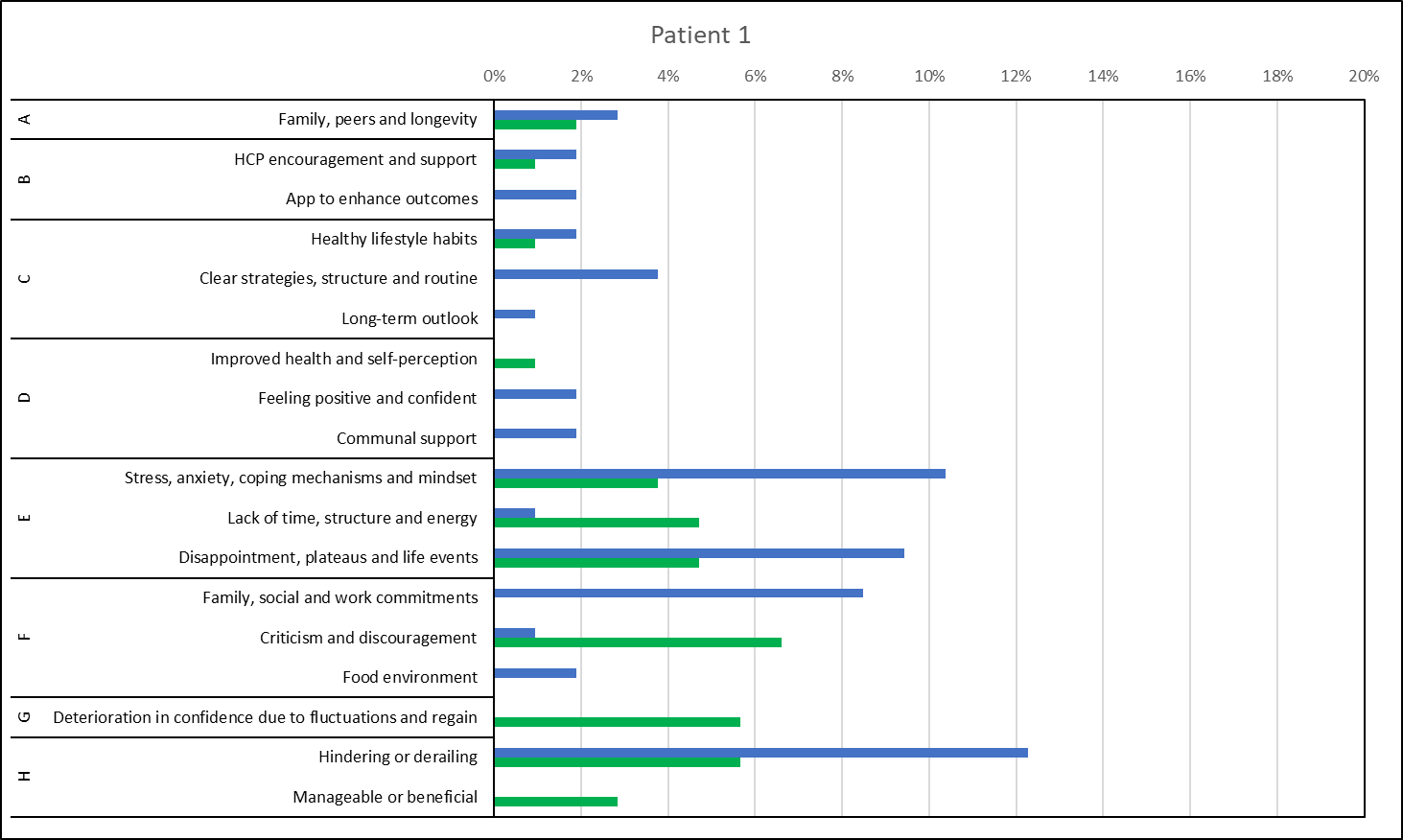


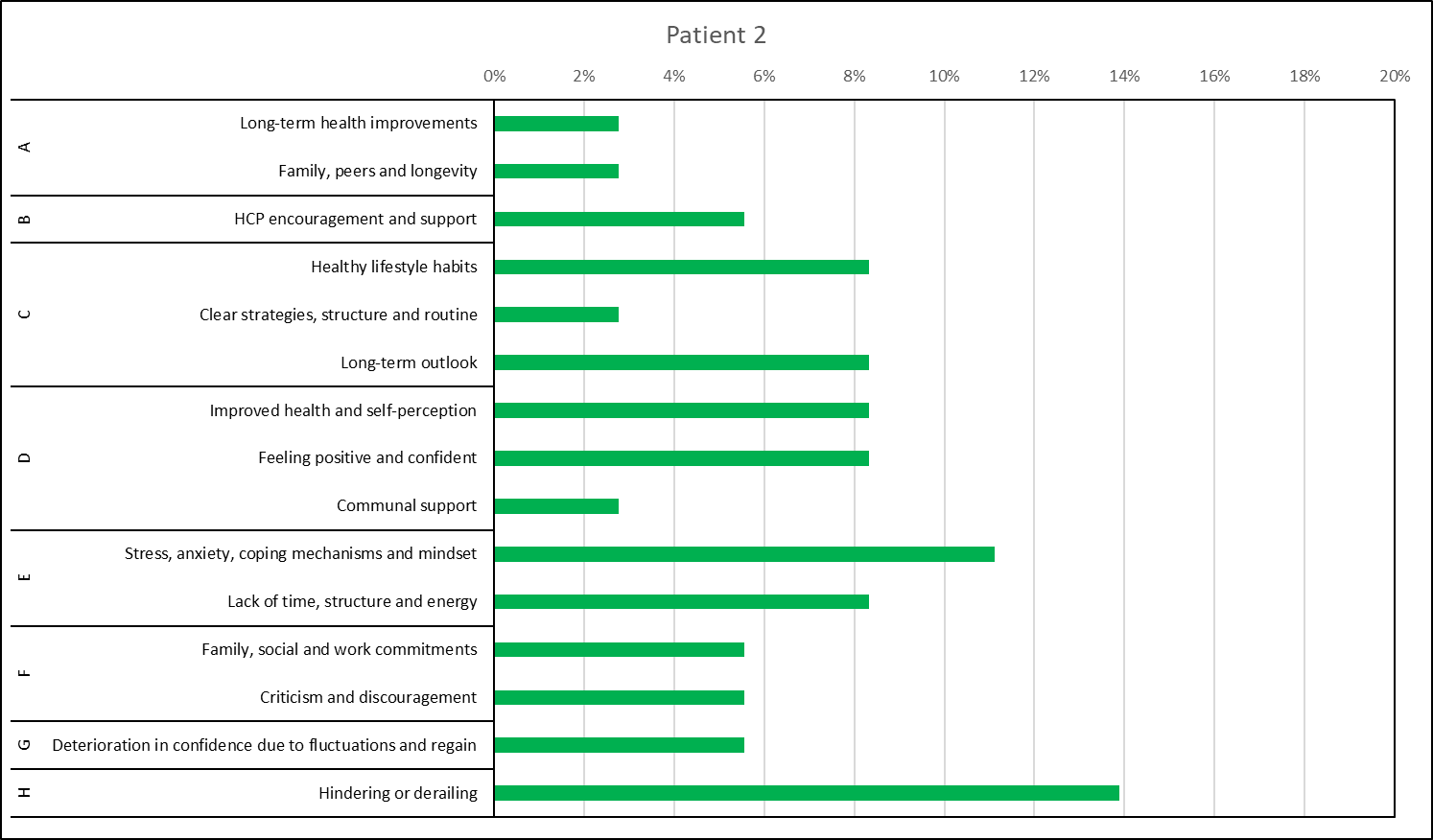


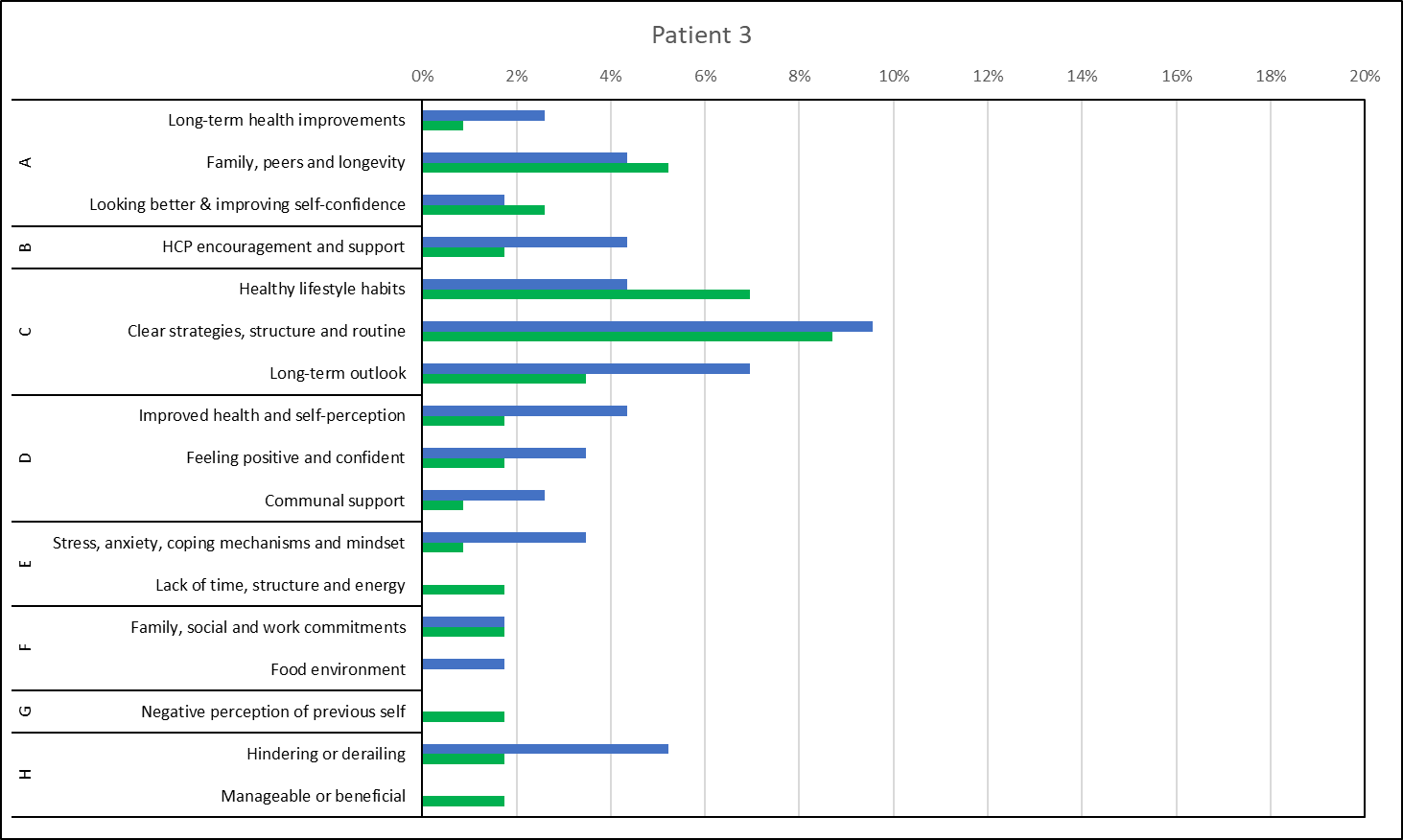


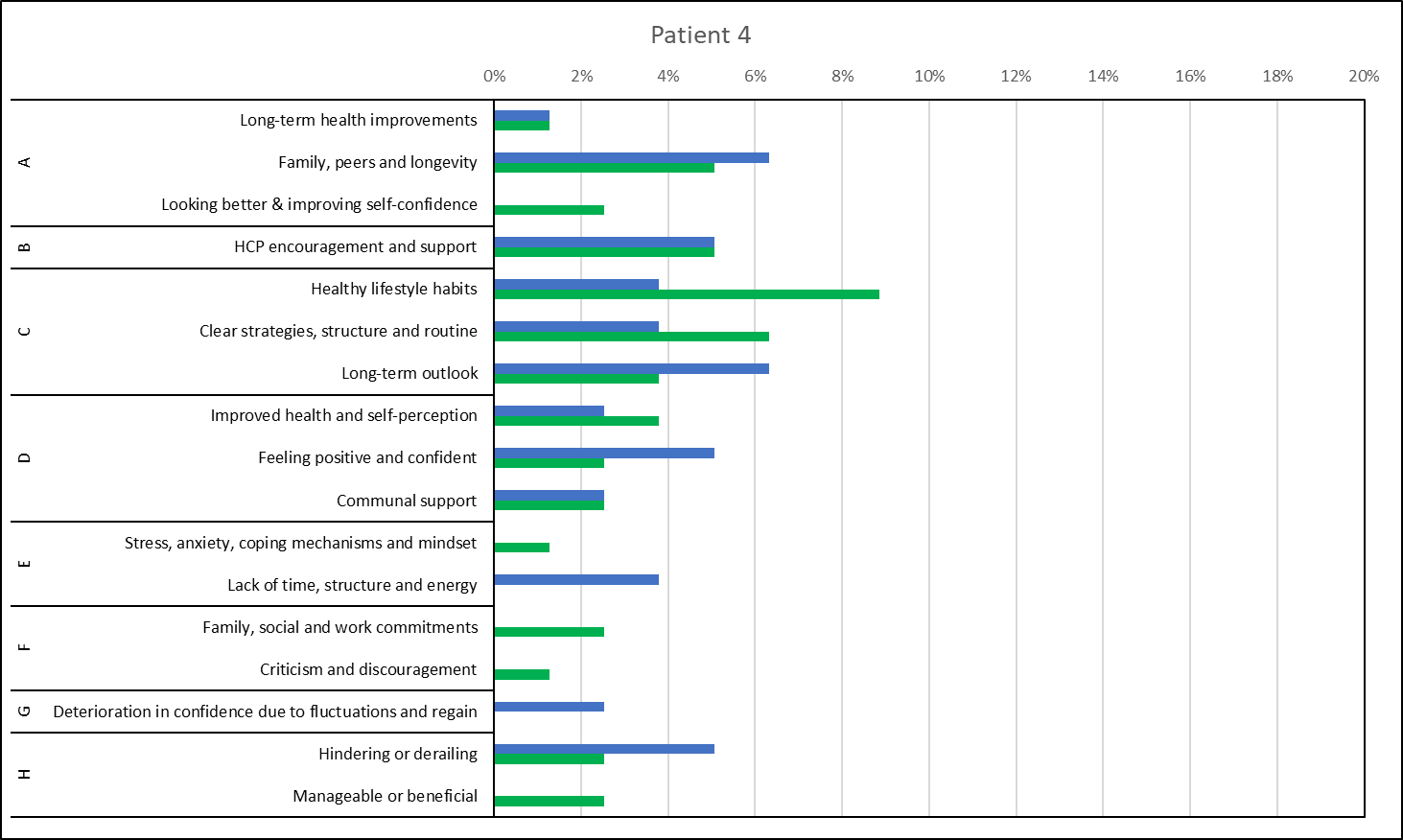


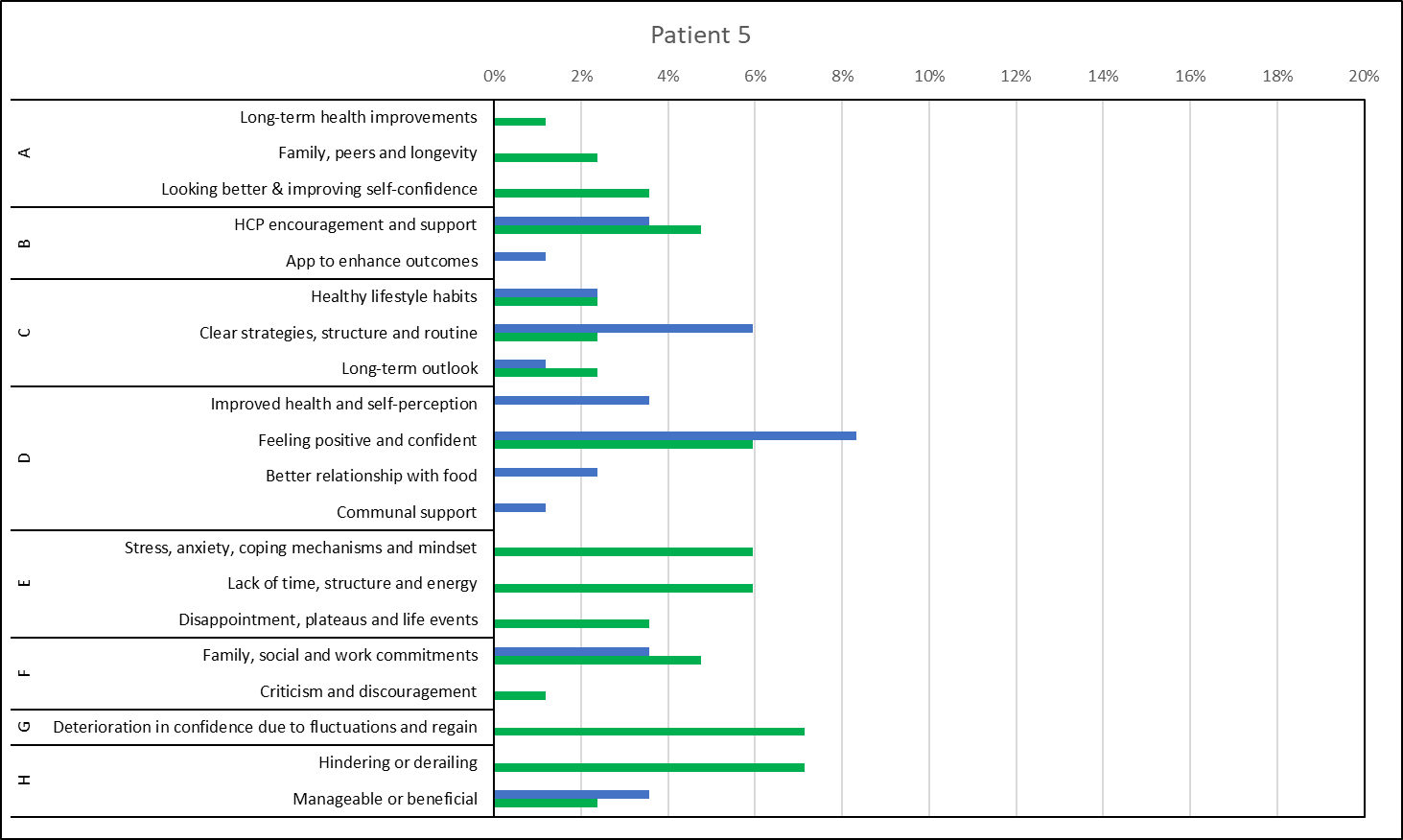


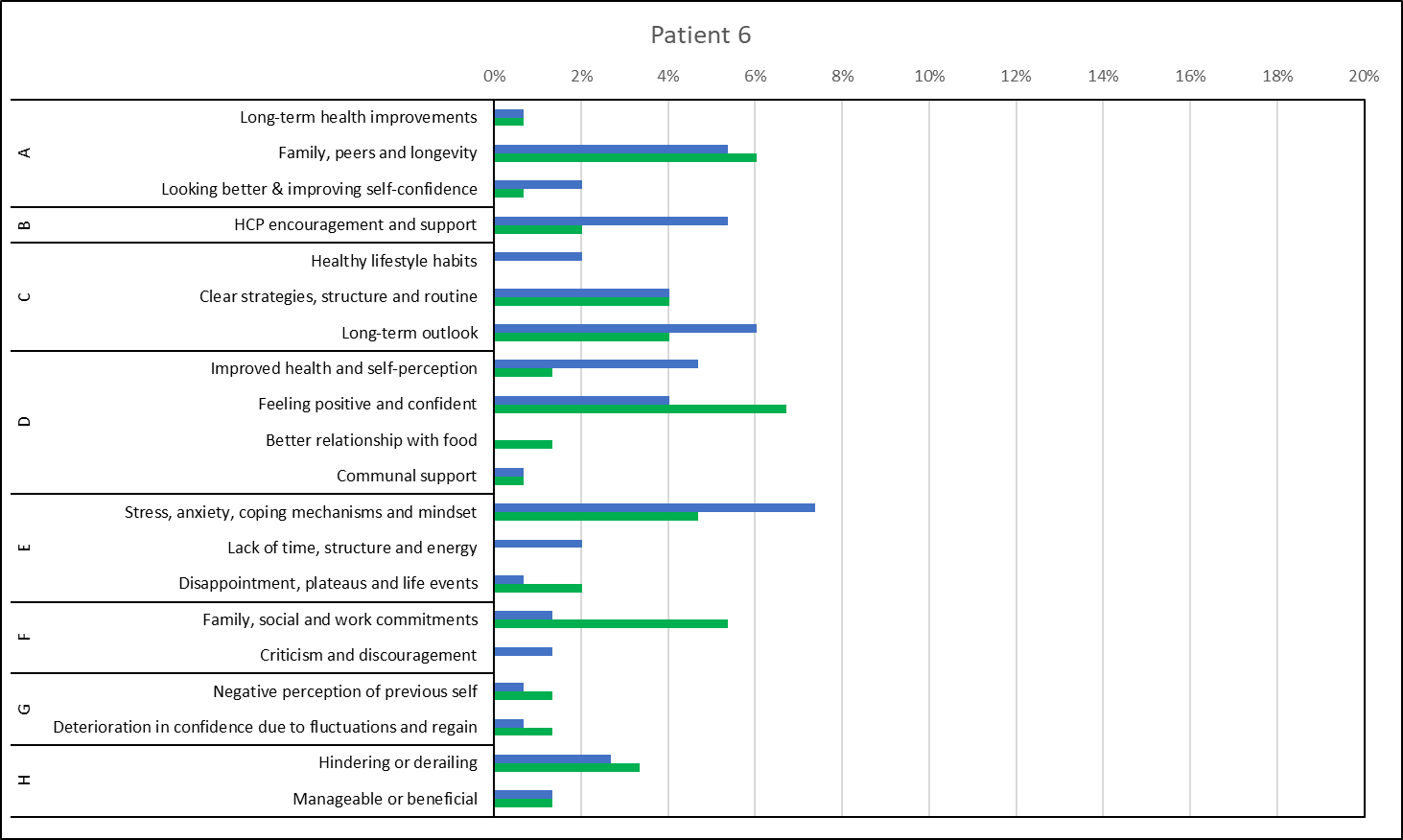


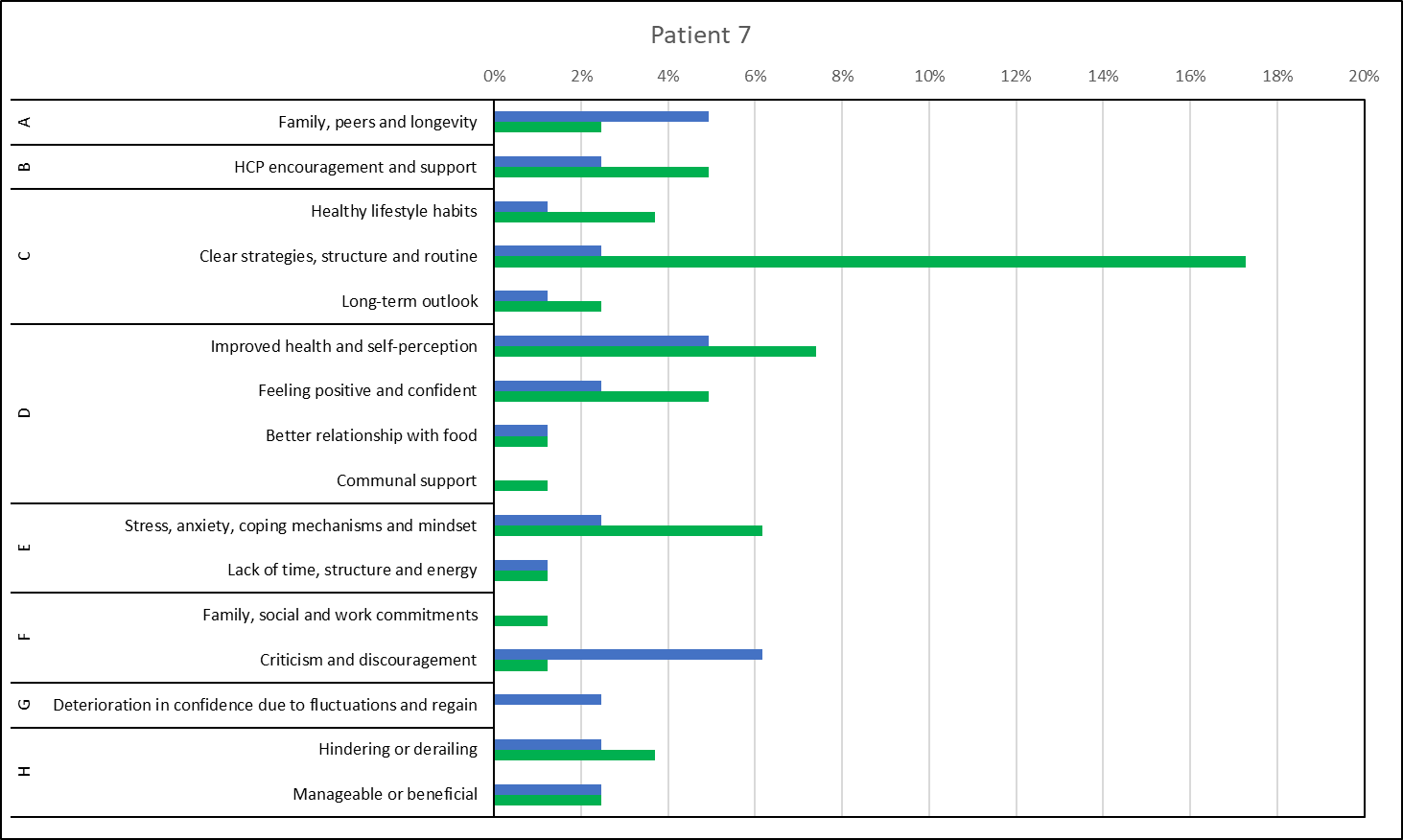


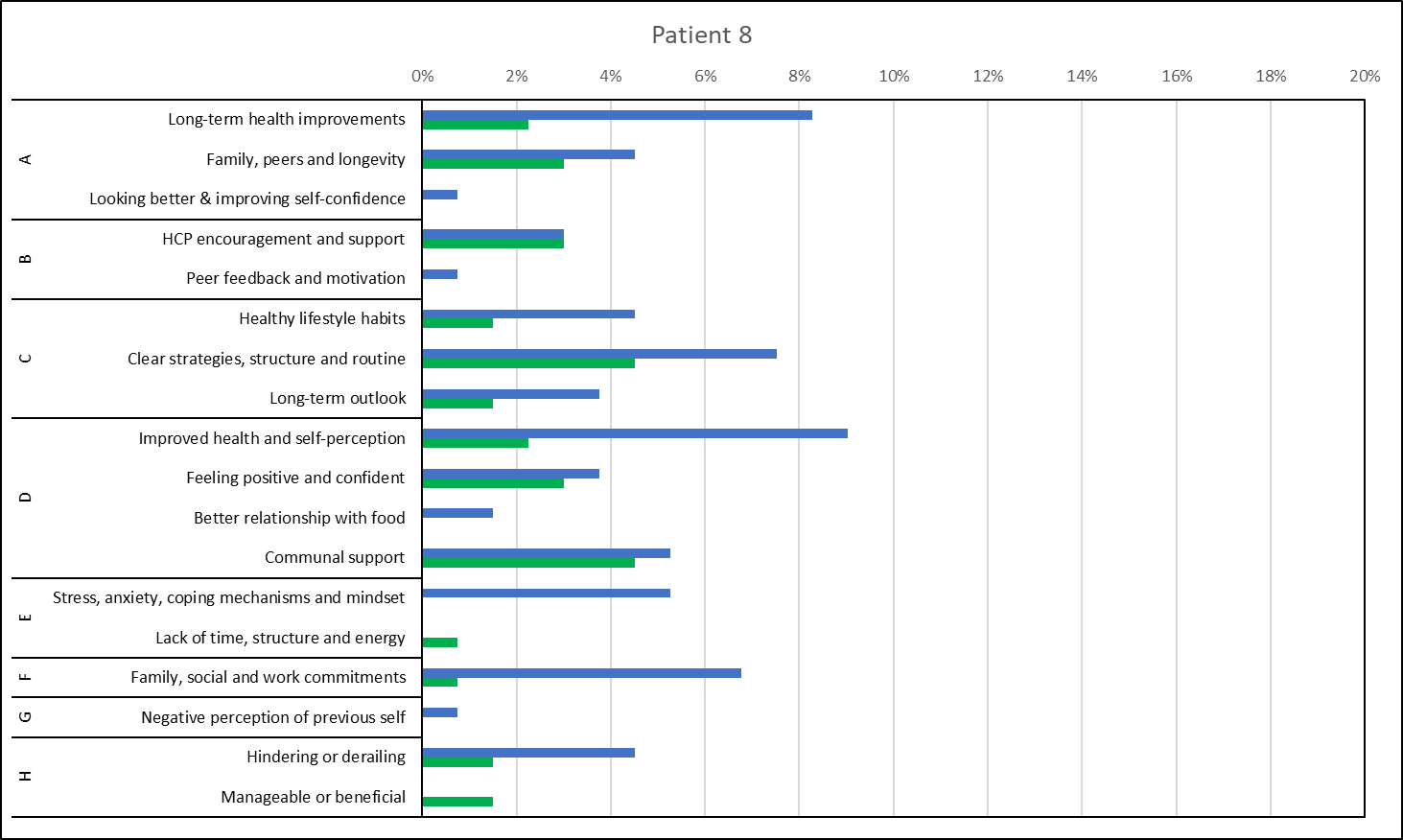


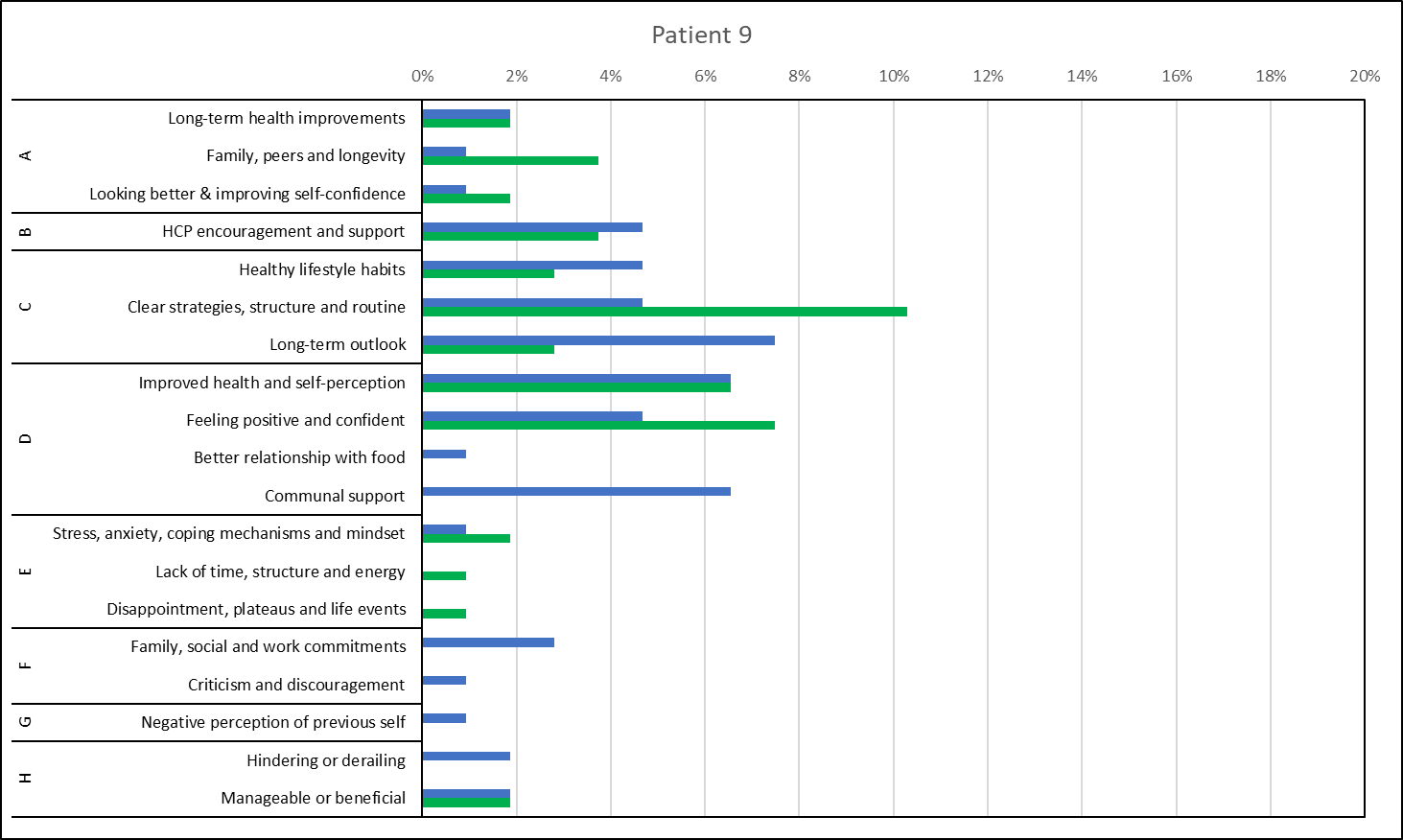


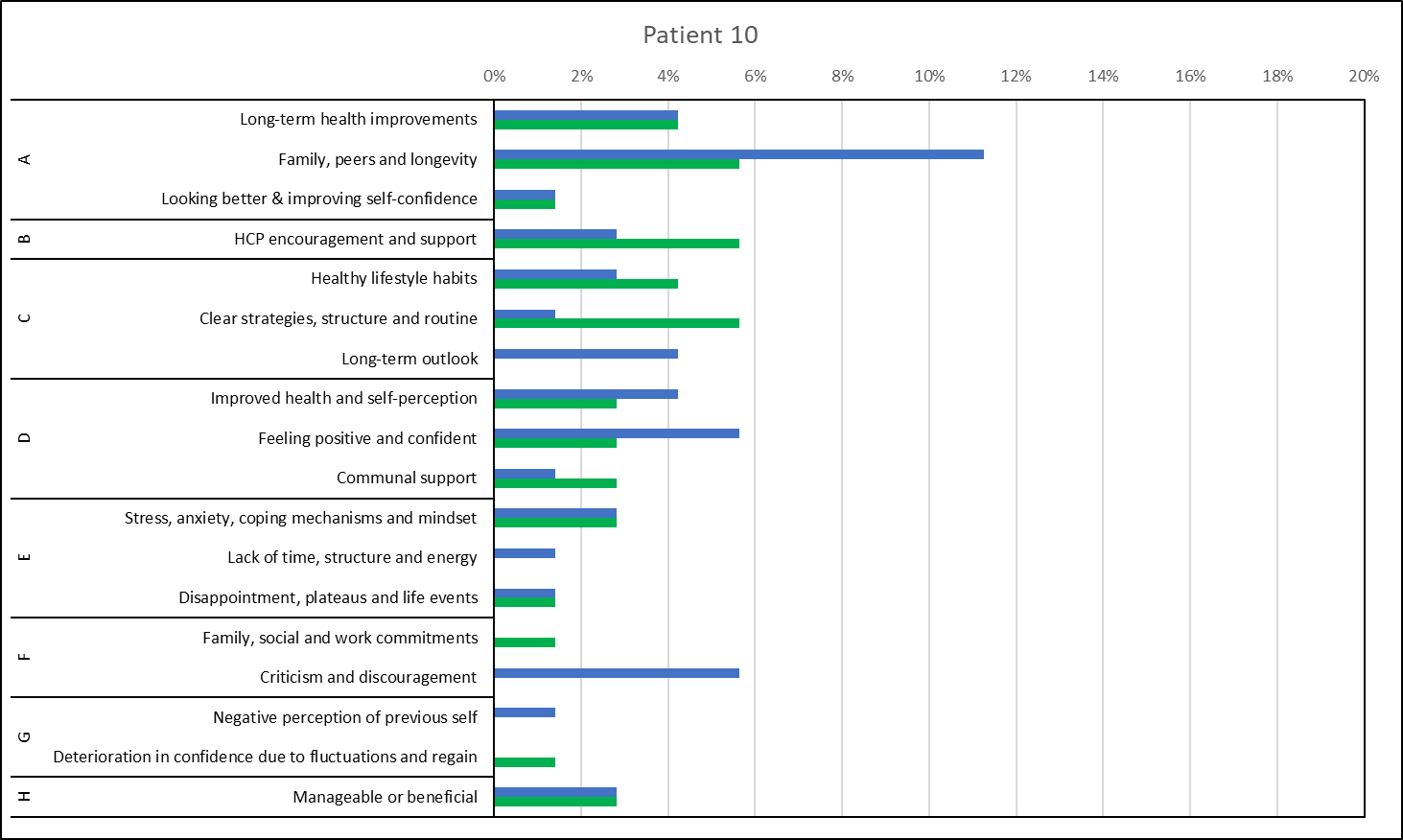


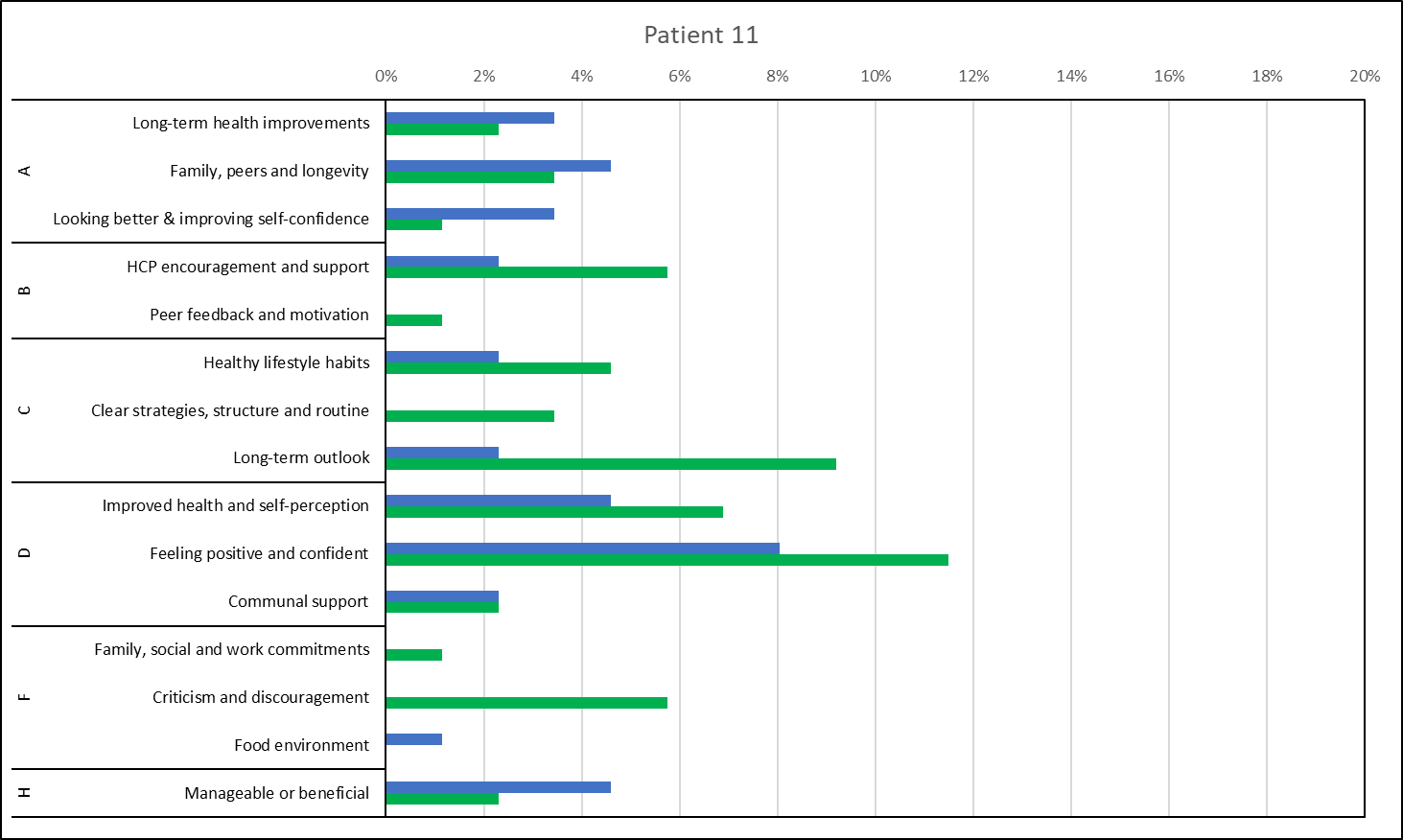


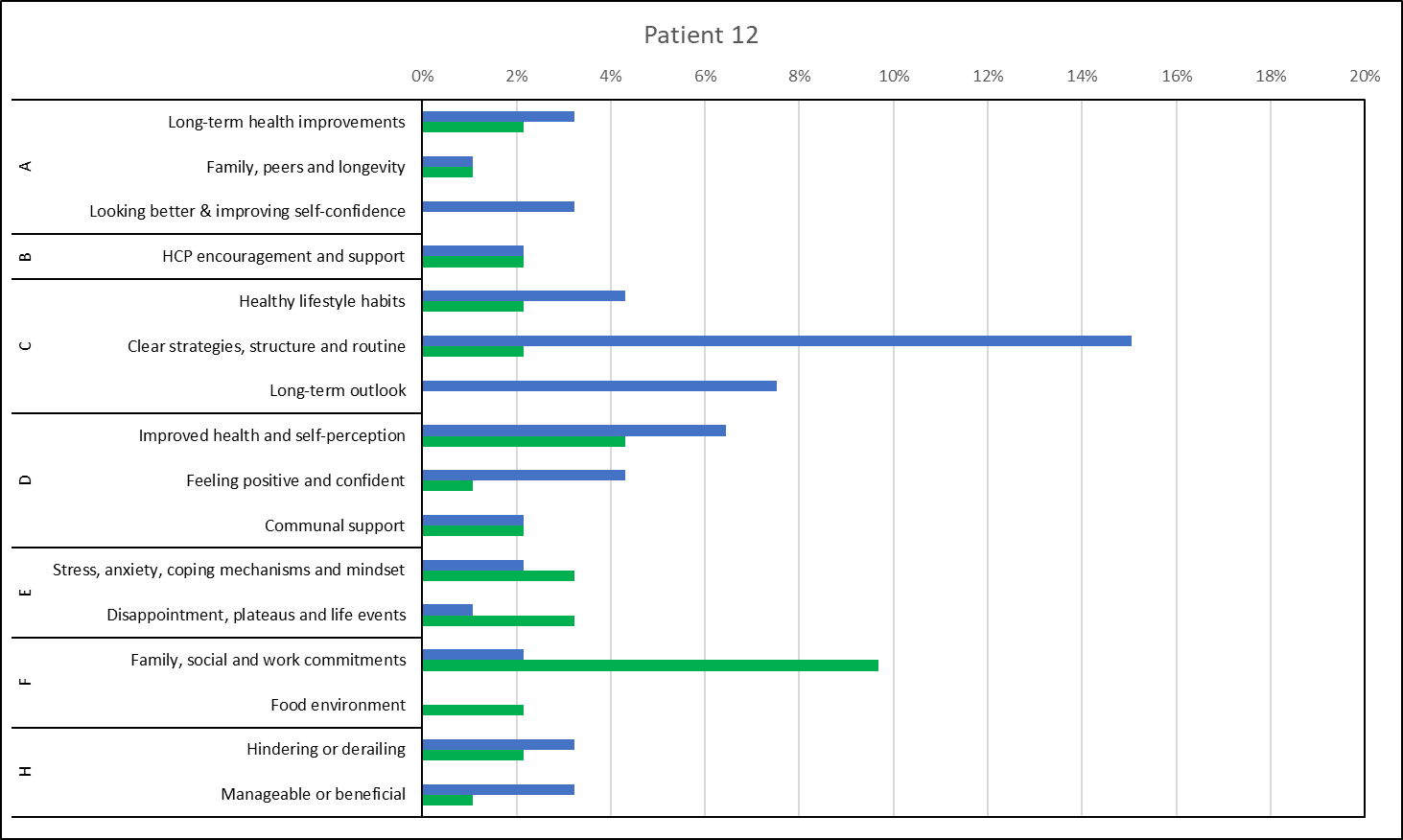


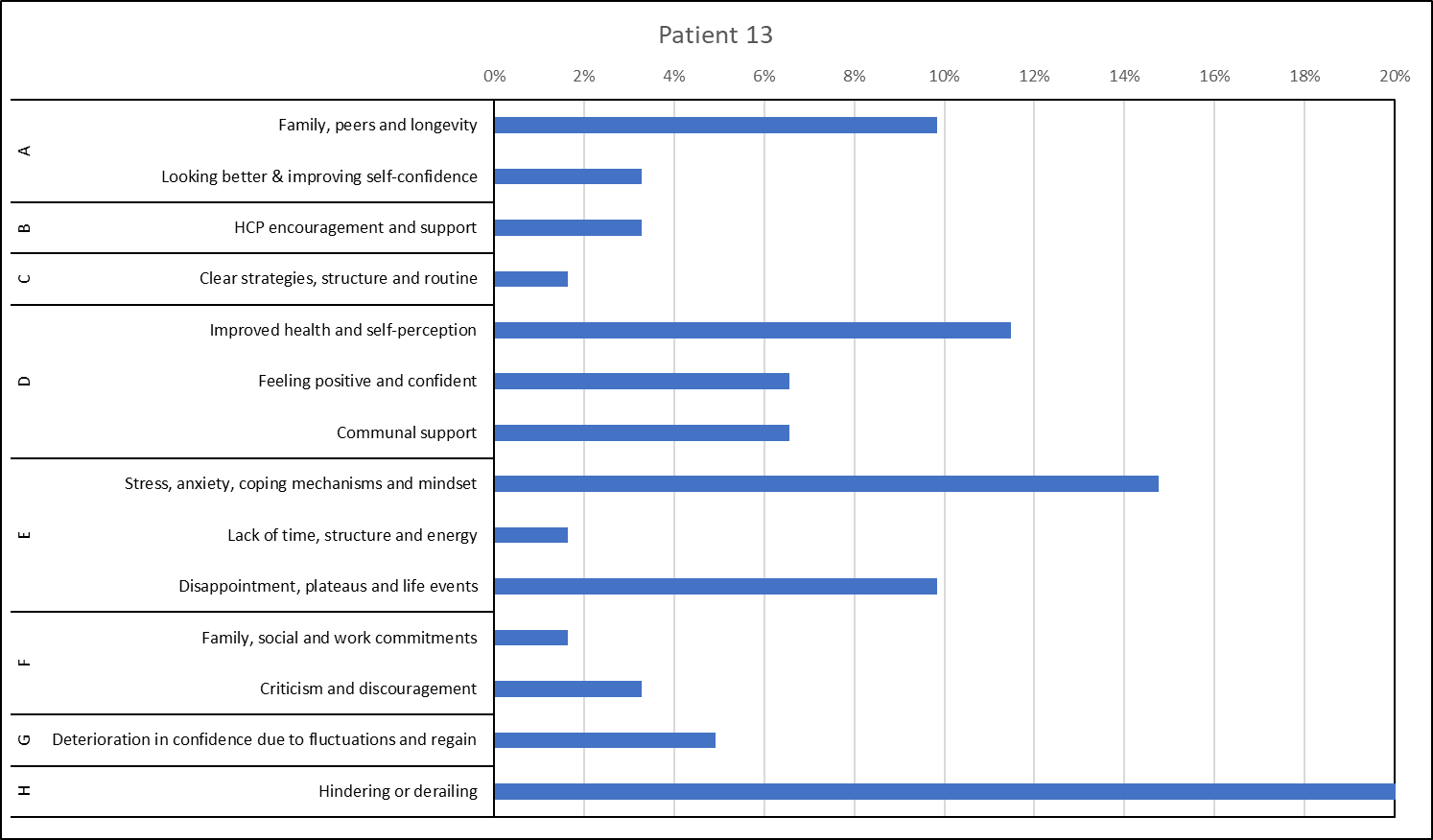


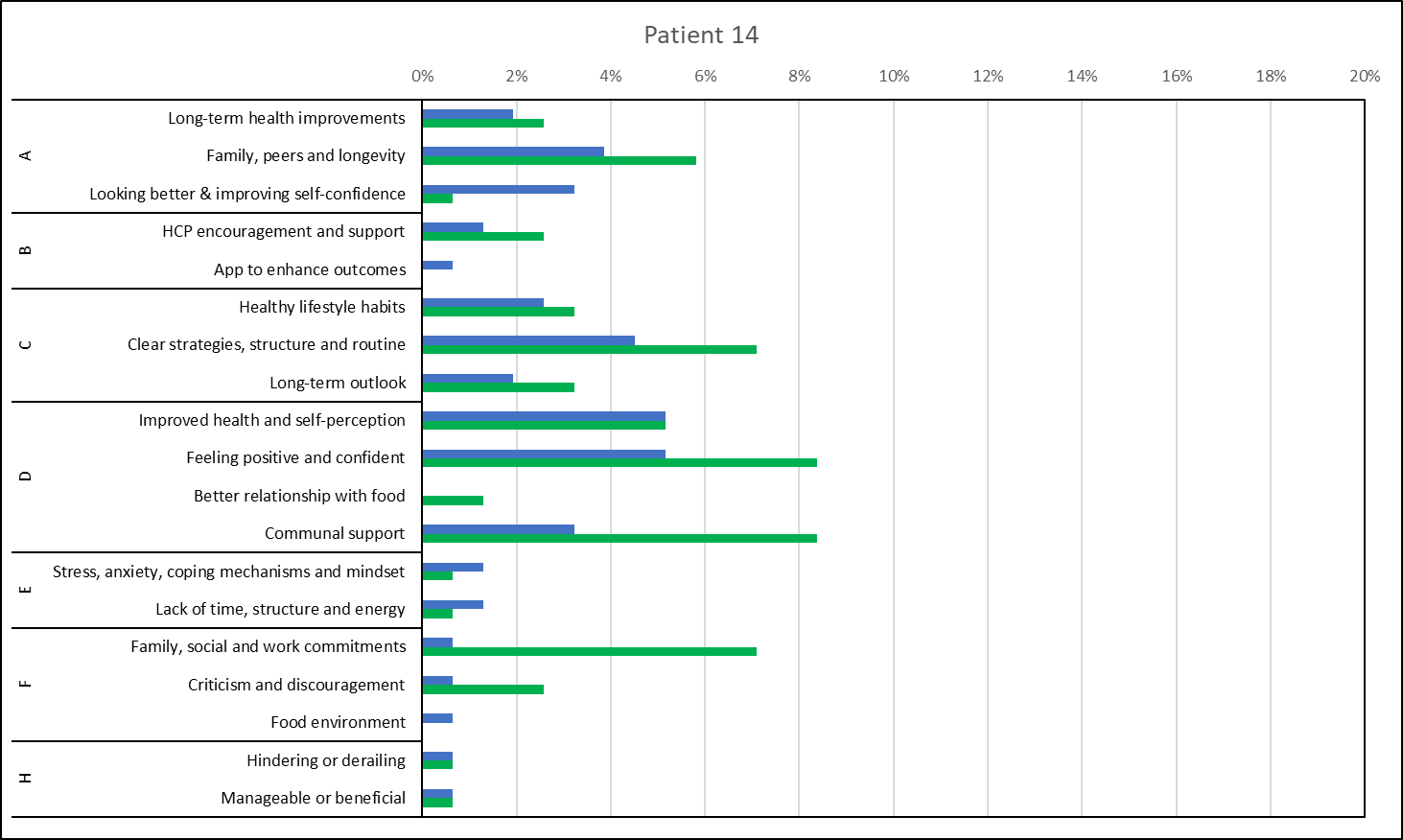


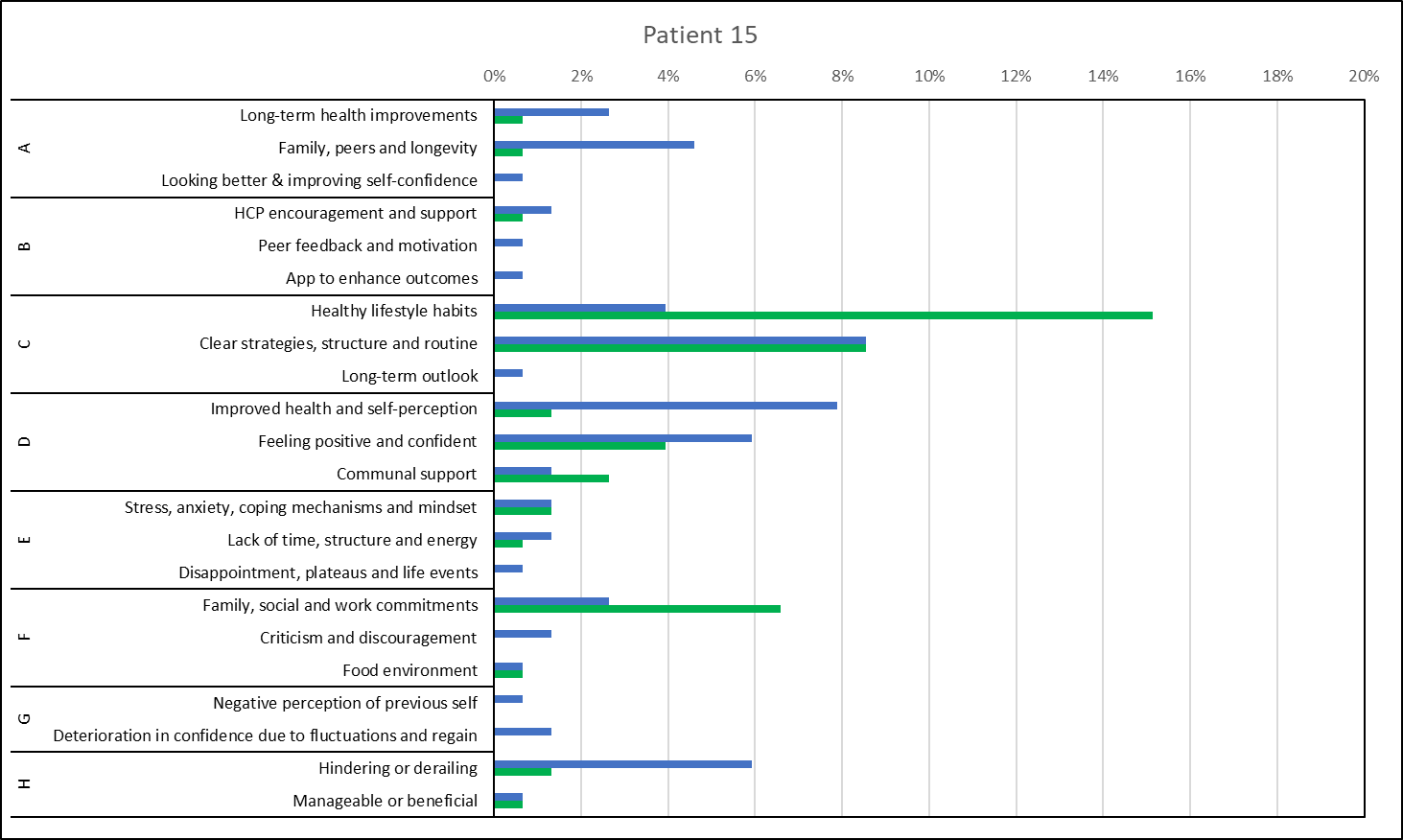


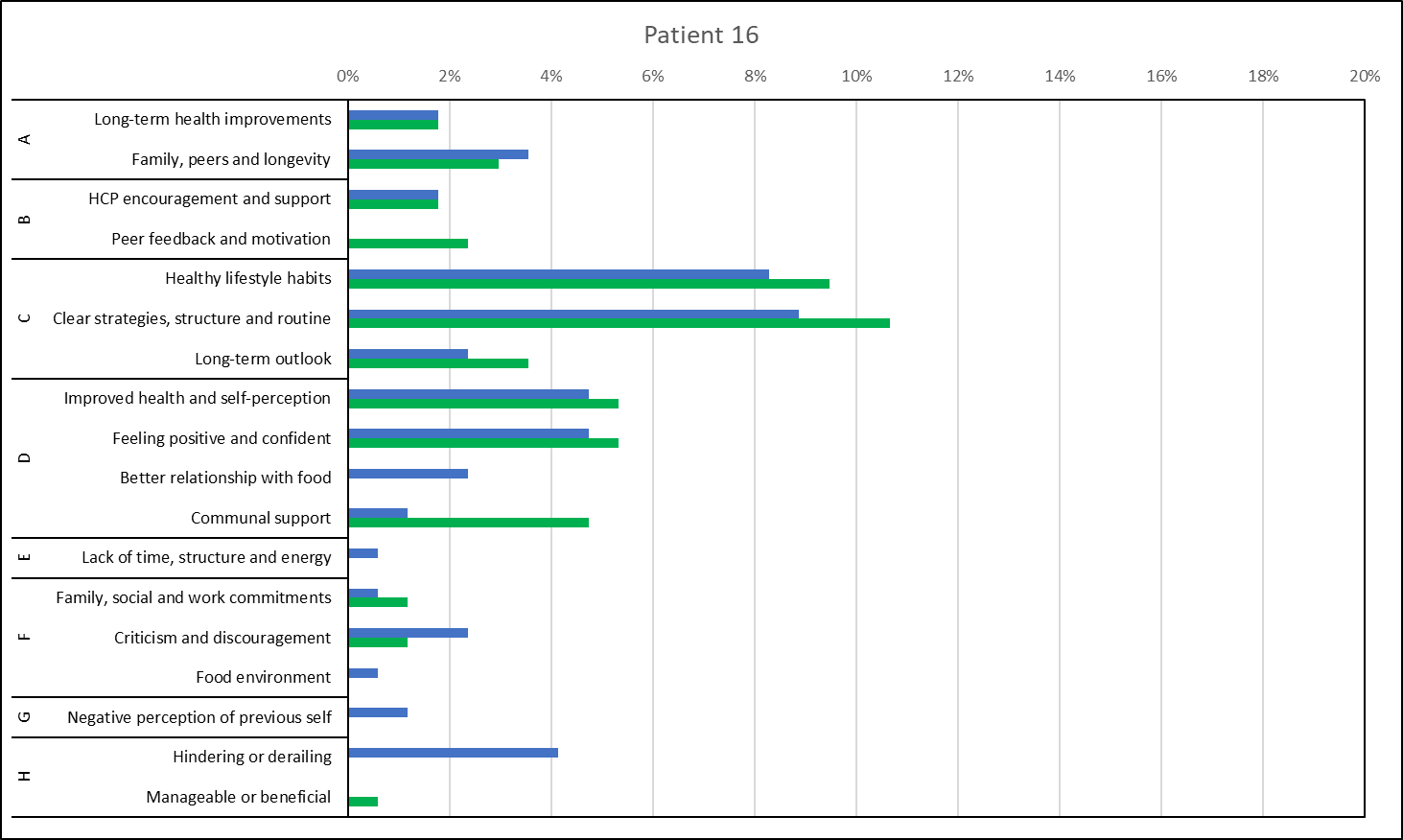


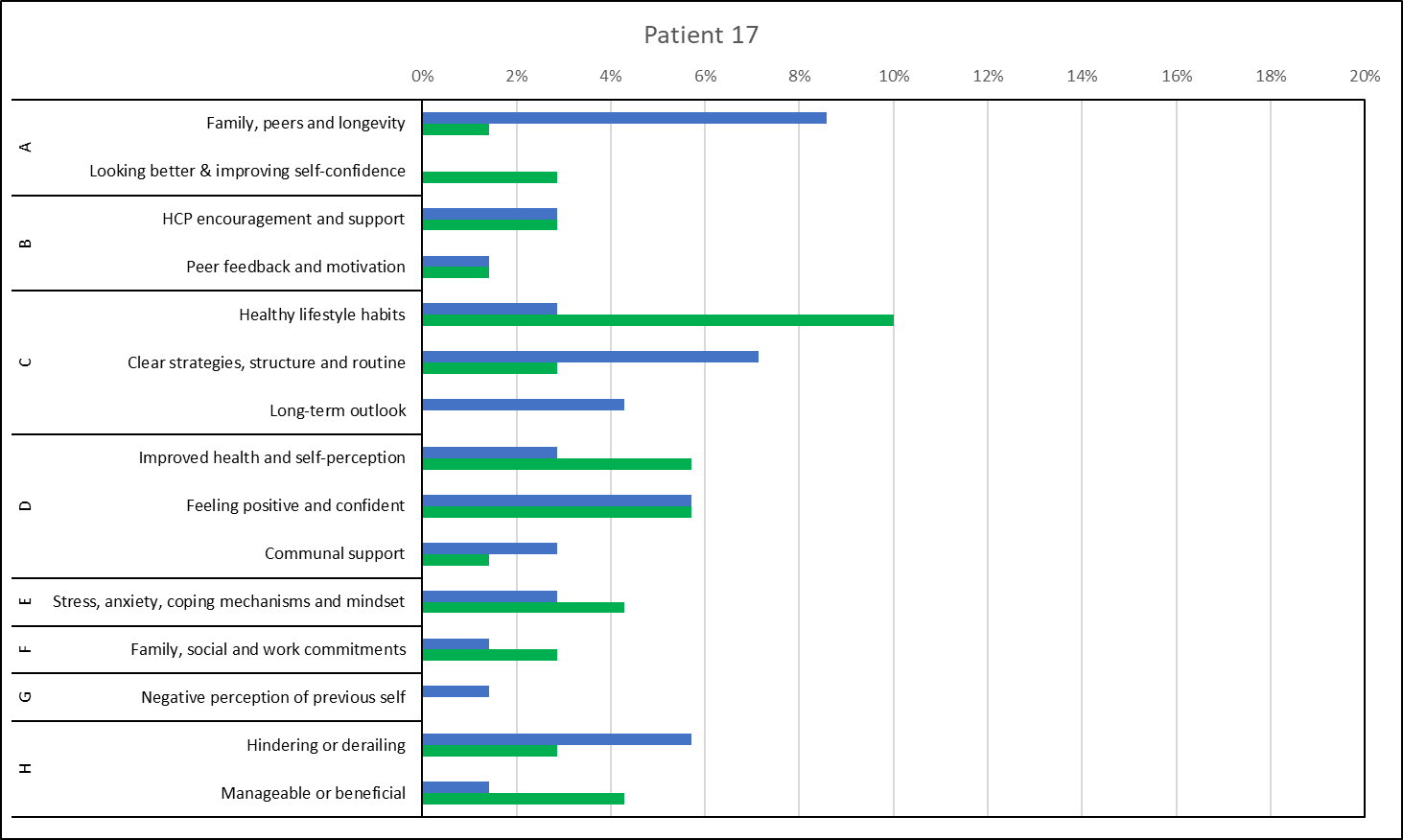


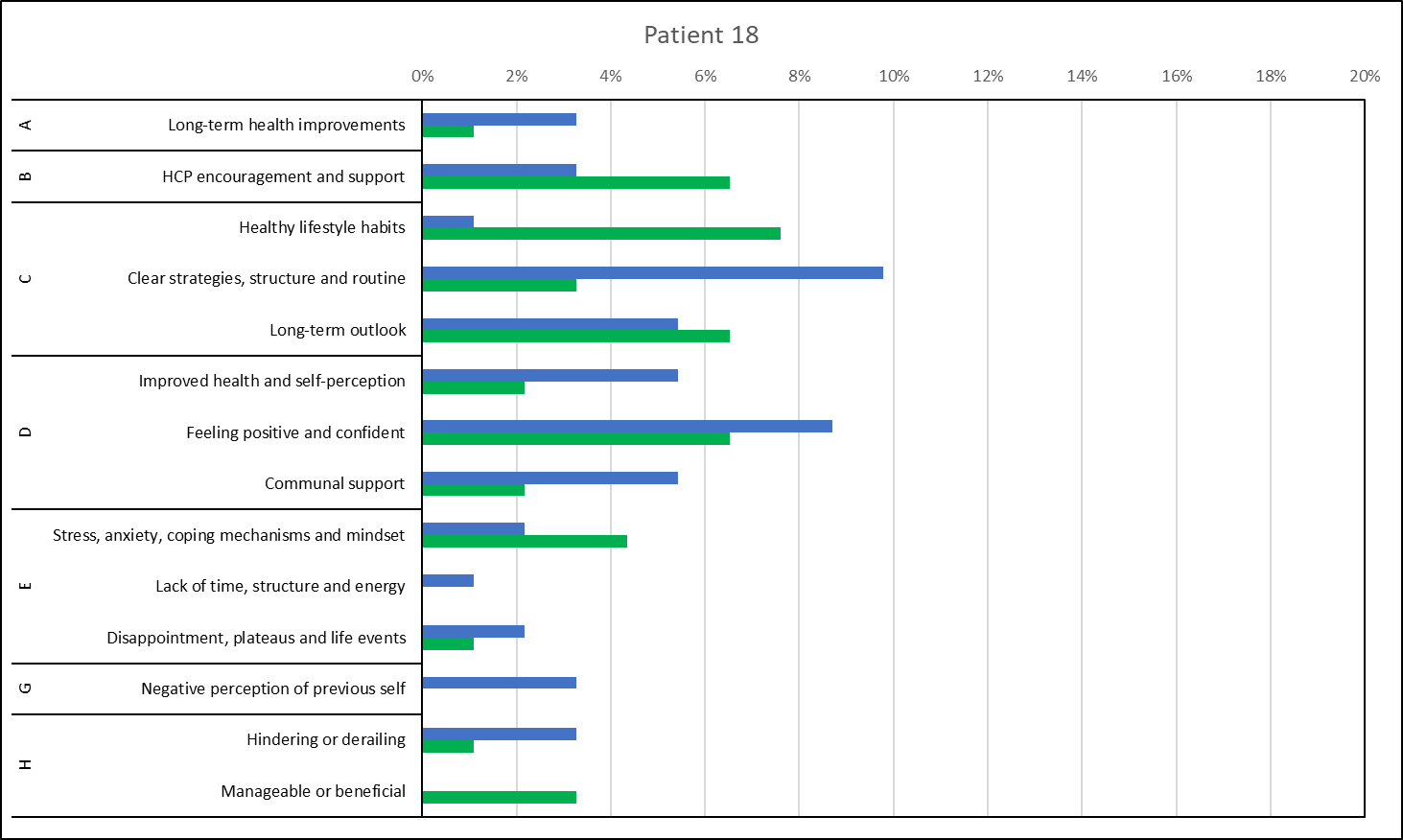


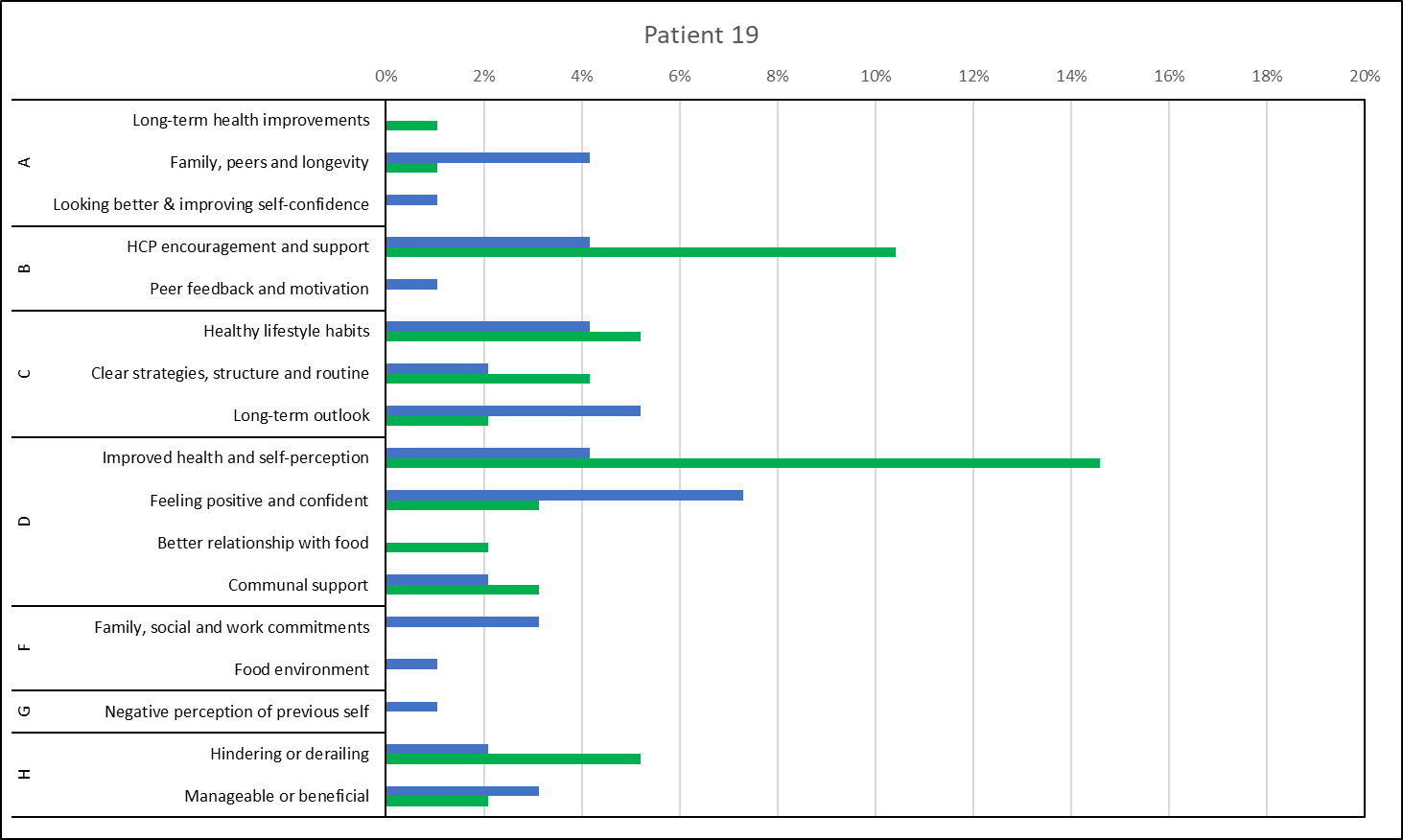


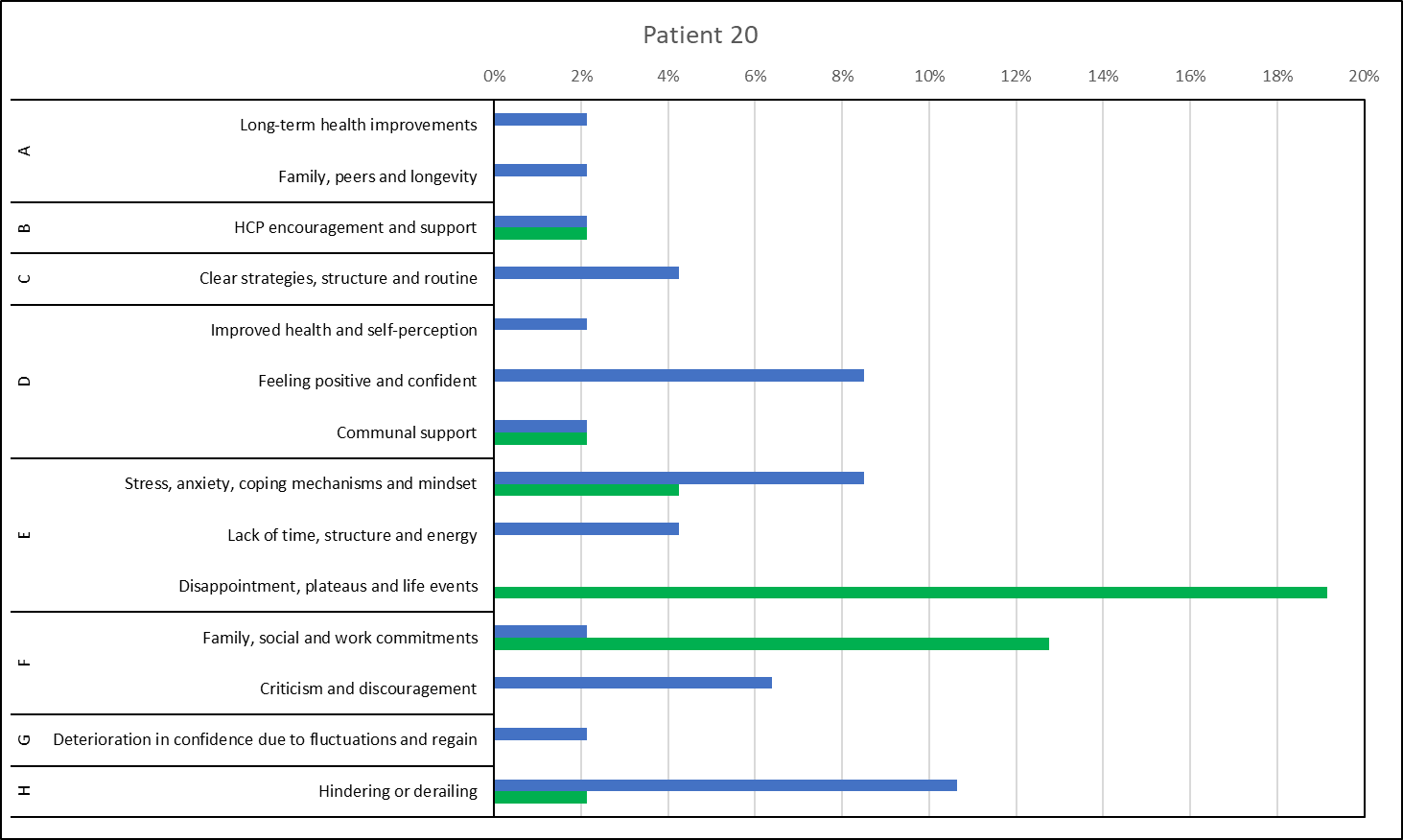


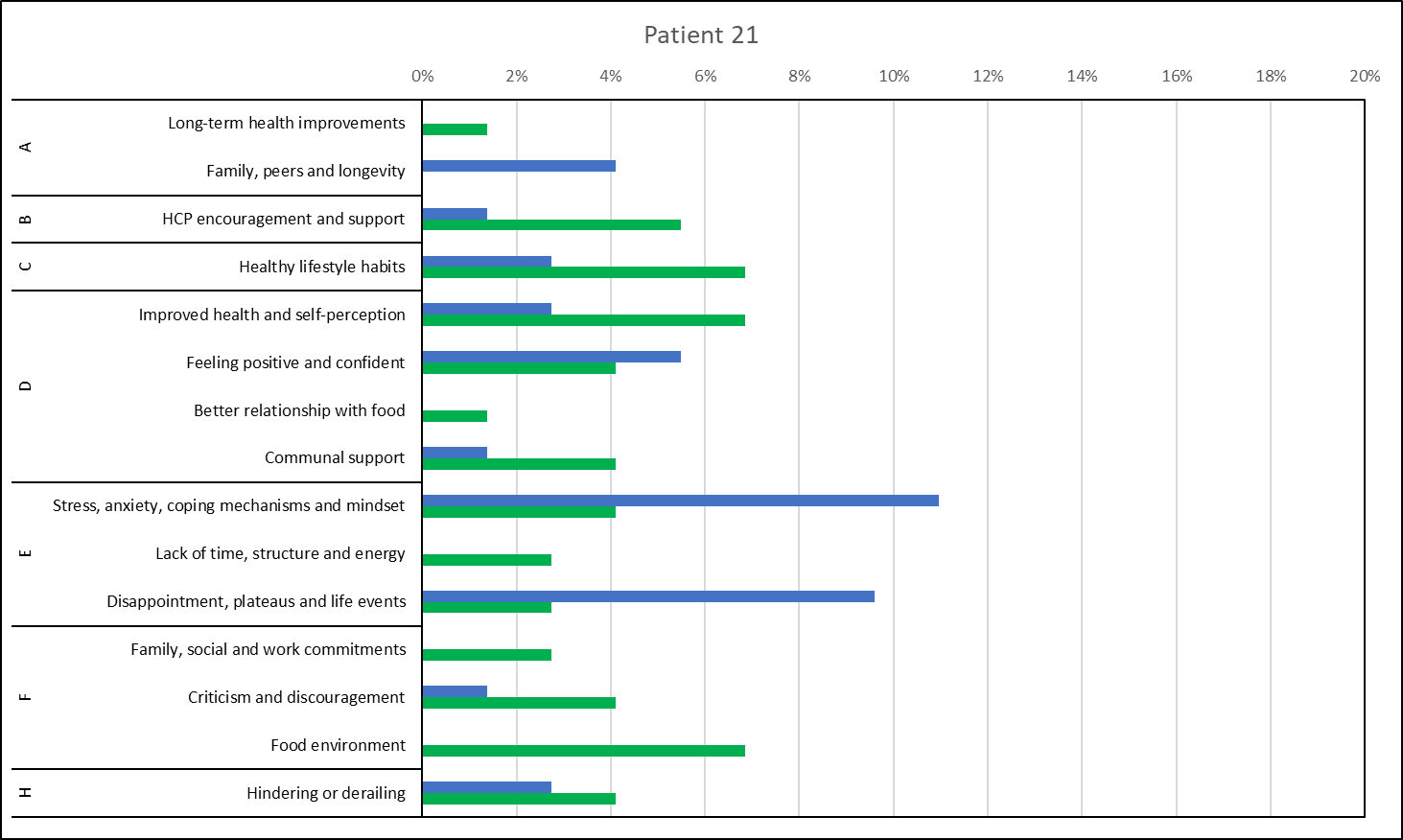

Supplement: Supplemental Material [file ZQHW_A_2276576_SM8692.docx]
